# Distinct Evolutionary Trajectories of Neuronal and Hair Cell Nicotinic Acetylcholine Receptors

**DOI:** 10.1093/molbev/msz290

**Published:** 2019-12-10

**Authors:** Irina Marcovich, Marcelo J Moglie, Agustín E Carpaneto Freixas, Anabella P Trigila, Lucia F Franchini, Paola V Plazas, Marcela Lipovsek, Ana Belén Elgoyhen

**Affiliations:** 1 Instituto de Investigaciones en Ingeniería Genética y Biología Molecular “Dr. Héctor N. Torres” (INGEBI), Consejo Nacional de Investigaciones Científicas y Técnicas (CONICET), Buenos Aires, Argentina; 2 Instituto de Farmacología, Facultad de Medicina, Universidad de Buenos Aires, Buenos Aires, Argentina; 3 Centre for Developmental Neurobiology, King’s College London, Institute of Psychiatry, Psychology and Neuroscience, Guy’s Campus, London, United Kingdom

**Keywords:** nicotinic receptors, molecular evolution, hair cells

## Abstract

The expansion and pruning of ion channel families has played a crucial role in the evolution of nervous systems. Nicotinic acetylcholine receptors (nAChRs) are ligand-gated ion channels with distinct roles in synaptic transmission at the neuromuscular junction, the central and peripheral nervous system, and the inner ear. Remarkably, the complement of nAChR subunits has been highly conserved along vertebrate phylogeny. To ask whether the different subtypes of receptors underwent different evolutionary trajectories, we performed a comprehensive analysis of vertebrate nAChRs coding sequences, mouse single-cell expression patterns, and comparative functional properties of receptors from three representative tetrapod species. We found significant differences between hair cell and neuronal receptors that were most likely shaped by the differences in coexpression patterns and coassembly rules of component subunits. Thus, neuronal nAChRs showed high degree of coding sequence conservation, coupled to greater coexpression variance and conservation of functional properties across tetrapod clades. In contrast, hair cell α9α10 nAChRs exhibited greater sequence divergence, narrow coexpression pattern, and great variability of functional properties across species. These results point to differential substrates for random change within the family of gene paralogs that relate to the segregated roles of nAChRs in synaptic transmission.

## Introduction

The superfamily of pentameric ligand-gated ion channels (LGIC) has an extended evolutionary history and is present in all three life domains (Tasneem et al. 2005; Dent 2006; Jaiteh et al. 2016). Extant members of the superfamily include eukaryote *Cys*-loop receptors that respond to acetylcholine (ACh), γ-aminobutyric acid (GABA), glycine or serotonin, invertebrate LGICs that respond to GABA, glutamate, ACh, serotonin, and histamine, as well as prokaryote pH and GABA sensitive receptors, among others (Cully et al. 1994; Karlin 2002; Tasneem et al. 2005; Dent 2006; Hilf and Dutzler 2009; Hervé Thany 2010; Spurny et al. 2012). Nicotinic acetylcholine receptors (nAChRs) are a major branch of the *Cys*-loop LGIC superfamily (Karlin 2002; Corringer et al. 2012). To date, 17 different nAChR subunits have been described in the main vertebrate clades: α1–α10, β1–β4, δ, ε, and γ (Karlin 2002; Spurny et al. 2012). These paralogous genes are proposed to derive from five paralogons, with the entire complement of extant subunits present in the vertebrate ancestor (Pedersen et al. 2019). Vertebrate nAChRs are nonselective cation channels that participate in numerous processes, most prominently neuromuscular junction (Steinbach 1989; Kalamida et al. 2007), inner ear efferent (Elgoyhen and Katz 2012), and neuronal (Dani and Bertrand 2007; Zoli et al. 2015) synaptic transmission.

Functional nAChRs are homomeric, comprising five identical subunits, or heteromeric, formed by at least two different subunits (Karlin 2002; Millar and Gotti 2009; Zoli et al. 2015). The rules that govern the combinatorial assembly of functional nAChRs are complex and for the most part unknown. Some nAChR subunits can combine with other numerous, albeit specific, subunits. In contrast, some subunits can only form functional receptors with a limited subset (Karlin 2002; Millar and Gotti 2009). This segregation has given rise to subgroups of vertebrate nAChRs named for their initially described tissue of origin and main functional location. Thus, neuronal nAChRs are formed by multiple combinations of α2–α7 (and α8 in nonmammals) and β2–β4 subunits, comprising a wide combinatorial range with alternative stoichiometries (Millar and Gotti 2009; Zoli et al. 2015, 2018). Muscle receptors show tighter coassembly rules. They have a typical α1_2_β1γδ (or α1_2_β1εδ) stoichiometry and do not coassemble with nonmuscle subunits (Mishina et al. 1986; Millar and Gotti 2009). Finally, the hair cell nAChR has a very strict coassembly constraint, being formed exclusively by α9 and α10 subunits (Elgoyhen et al. 2001; Sgard et al. 2002; Scheffer et al. 2007). Although α9 subunits can form functional homomeric receptors (Elgoyhen et al. 1994; Lipovsek et al. 2014), these are unlikely to play a major role in inner ear hair cells in vivo (Vetter et al. 2007). Although the α9 and α10 subunits were initially classified as a neuronal, the α9α10 receptor does not appear to be functionally present in the brain (Morley et al. 2018). A consequence of the differences in coassembly rules between the three subgroups of nAChRs is that, although muscle cells can toggle between at least two receptor variants and neurons are capable of expressing a great diversity of nAChRs, hair cells express only one type.

The complement of nAChR subunits is highly conserved across vertebrates and even more so in tetrapods (Dent 2006; Pedersen et al. 2019). This suggests a high gene family-wide negative selection pressure for the loss of paralogs and highlights the functional relevance of each individual subunit across the vertebrate clade. However, given the major differences in coexpression patterns and coassembly rules that distinguish the subgroups of nAChRs, in particular the contrast between neuronal and hair cell receptors, distinct evolutionary trajectories most likely took place in different members of the family. On the one hand, if only one (functionally isolated) receptor is expressed by a given cell type, then selection pressure could have acted on stochastic changes of the coding sequence of component subunits that altered receptor function. Such changes would not have resulted in deleterious effects on other nAChRs given the restricted expression pattern of the subunits. The aforesaid process could have dominated the evolutionary history of the hair cell α9α10 receptor. On the other hand, widely expressed subunits that coassemble into functional receptors with multiple others have been most probably subjected to strong negative selection pressure. Changes in the coding sequence that lead to changes in functional properties may have had deleterious effects on alternative receptor combinations expressed in different cell types. In this case, functional diversification could have arisen from stochastic changes in the expression patterns of receptor subunits, resulting in a given cell changing the subtype of receptor it expresses, while preserving individual subunit functionality. Such processes could have dominated the evolutionary history of neuronal nAChRs. These contrasting theoretical scenarios bring forward three predictions for the evolutionary history of nAChRs across the vertebrate clade. First, isolated (hair cell) subunits may show coding sequence divergence, whereas widespread (neuronal) subunits a high degree of coding sequence conservation. Second, isolated subunits may show low coexpression variation, whereas widespread subunits a great variability in coexpression patterns. Finally, isolated receptors may present divergent functional properties across species resulting from changes in coding sequences, whereas widespread receptors may show highly conserved functional properties when formed by the same subunits.

To test these predictions, we studied the molecular evolution and the variability in expression pattern, coupled to coassembly potential, of vertebrate nAChR subunits. Furthermore, we performed a comprehensive comparative analysis of the functional properties of the hair cell (α9α10) and the two main neuronal (α7 and α4β2) nAChRs from three representative species of tetrapods. We present strong evidence supporting the notion that, within the family of paralog genes coding for nAChR subunits, receptors belonging to different subgroups (hair cell or neuronal) underwent different evolutionary trajectories along the tetrapod lineage that were potentially shaped by their different coexpression patterns and coassembly rules. We propose that it is the difference in the most probable substrate for random change and subsequent selection pressure that separate the contrasting evolutionary histories of nAChRs subgroups.

## Results

### Amino Acid Sequence Divergence and Coexpression Patterns Differentiate Neuronal from Hair Cell nAChR Subunits

**FIg. 1.**
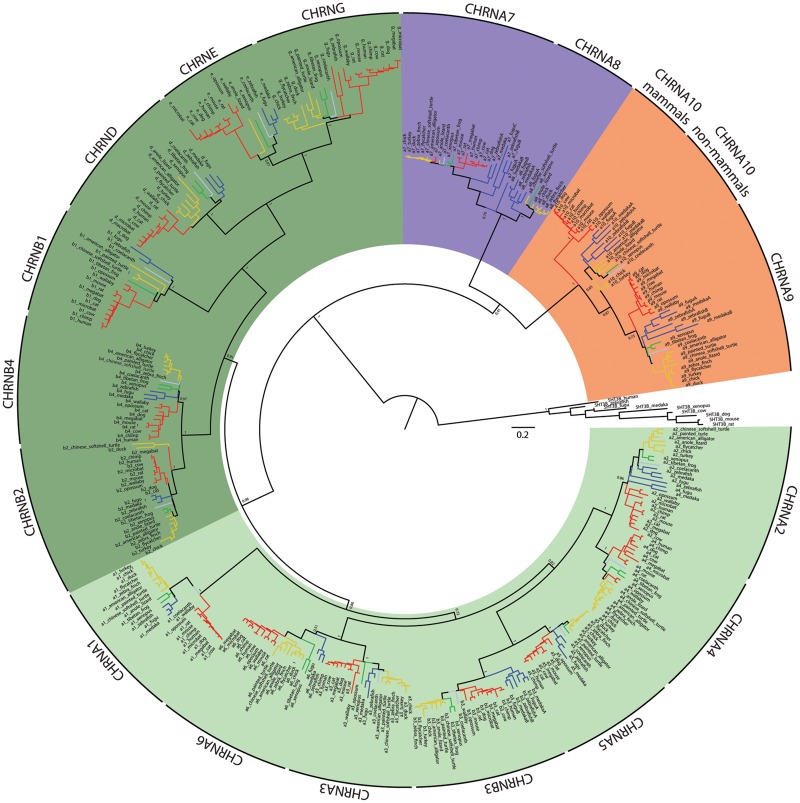
Phylogenetic tree of vertebrate nAChR subunits. Complete minimum evolution phylogenetic tree corresponding to the collapsed tree shown in figure 2*A*, obtained with variation rates among sites modeled by a gamma distribution. Red branches, mammals; yellow branches, sauropsids; green branches, amphibians; blue branches, fish; light blue branches, coelacanth. Shadings denote the different groups of subunits: light greens, α subunits; dark green, non-α subunits; purple, α7-like subunits; orange, α9-like subunits. The tree was built using minimum evolution method and pairwise deletion for missing sites. The optimal tree with a sum of branch length of 47.32565515 is shown. For clarity, the percentage of replicate trees in which the associated taxa clustered together in the bootstrap test (1,000 replicates) are shown only next to the branches that separate different subunits. The tree is drawn to scale, with branch lengths in the same units as those of the evolutionary distances used to infer the tree.

**FIg. 2.**
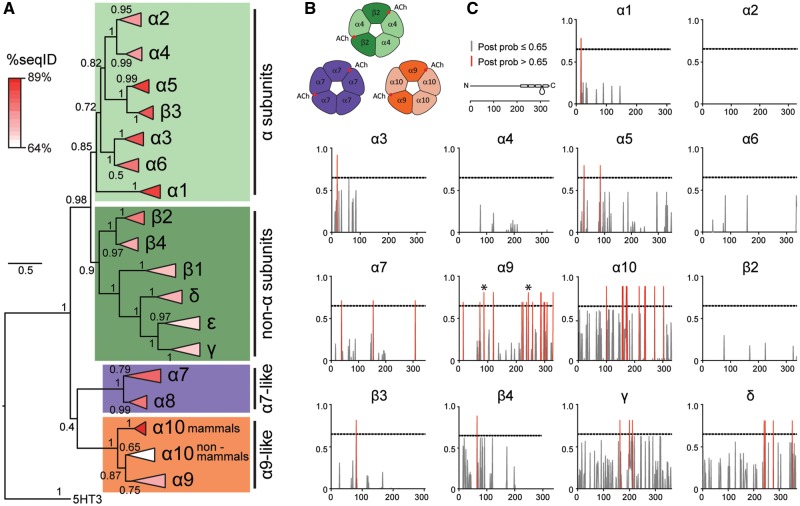
Hair cell nAChR subunits show greater sequence divergence than neuronal subunits. (*A*) Phylogenetic relationships between vertebrate nicotinic subunits. The branches corresponding to the same subunits of different species were collapsed up to the node at which one subunit separates from its closest neighbor. The complete tree is shown in figure 1. Triangles length denotes the divergence on sequence identity from the subunit node. Triangles were colored according to the average percentage of sequence identity between all pairs of sequences (%seqID, supplementary table S2, Supplementary Material online) within the branch. Shadings denote the different groups of subunits, as in figure 1: light greens, α subunits; dark green, non-α subunits; purple, α7-like subunits; orange, α9-like subunits. Numbers in branches indicate the bootstrap value obtained after 1,000 replicates. Scale bar indicates the number of amino acid substitutions per site. (*B*) Schematic diagrams of pentameric assemblies of neuronal and hair cell nAChRs. Red circles denote the positions of the ACh binding sites. (*C*) Posterior probabilities for type II functional divergence between mammalian and sauropsid clades, for each site along individual nAChR subunits. Gray lines, posterior probability ≤ 0.65. Red lines, posterior probability > 0.65. Diagram of a nAChR subunit extracellular, four transmembrane, and intracellular domains along amino acid position. * in α9 subunits plot, sites determinant of calcium permeability differences identified in Lipovsek et al. (2014).

The comparative analysis of gene paralogs provides the opportunity to test predictions about the evolutionary history of a gene family. To study the degree of conservation of coding sequences in subgroups of nAChRs, we performed an exhaustive evaluation of sequence divergence of vertebrate nAChR subunits. The analysis included amino acid sequences from 11 species of birds, reptiles, and amphibians, all groups that were notably underrepresented in previous work (Ortells and Lunt 1995; Tsunoyama and Gojobori 1998; Le Novère et al. 2002; Dent 2006; Franchini and Elgoyhen 2006; Lipovsek et al. 2012) and are of particular importance to help resolve differences between clades. Overall, we analyzed 392 sequences from 17 nAChR subunits belonging to 29 vertebrate species (supplementary table S1, Supplementary Material online, and fig. 1). Based on sequence identity, the family of nAChR subunits can be split into four groups: α, non-α, α7-like, and α9-like (figs. 1 and 2, supplementary fig. S1, Supplementary Material online, and Karlin [2002] and Millar and Gotti [2009]). α subunits are characterized by the presence of two consecutive cysteine residues in the ACh binding site. In the pentameric assembly, α subunits provide the principal component of the ACh binding domain and they require the coassembly with non-α subunits to form functional receptors. Non-α subunits lack the consecutive cysteines and contribute the complementary component of the ACh binding domain. α7-like subunits the possess consecutive cysteines and are capable of assembling homopentameric receptors, thus providing both the principal and complementary component of the ACh binding domain (figs. 1 and 2 and Karlin [2002]). Finally, α9-like subunits also have the consecutive cysteines and only form receptors with each other (Elgoyhen et al. 2001). As previously reported with smaller data sets (Franchini and Elgoyhen 2006; Lipovsek et al. 2012), the present extended analysis showed that α10 subunits are unique in presenting a segregated grouping of orthologs with nonmammalian α10 subunits as a sister group to all α9 subunits, and mammalian α10 subunits an outgroup to the α9/nonmammalian α10 branch (figs. 1 and 2). This may relate to the overall low %seqID of all vertebrate α10 subunits, coupled to high sequence conservation within individual clades (supplementary table S2, Supplementary Material online), together with the higher rate of nonsynonymous substitutions reported for the mammalian clade (Franchini and Elgoyhen 2006; Lipovsek et al. 2012). Next, we evaluated whether an accumulation of clade-specific amino acid changes was present in any nicotinic subunit that could point to clade-specific changes in protein function. To achieve this, we searched for site-specific evolutionary shifts in amino acid biochemical state between clades using the DIVERGE 3.0 software (Gu et al. 2013). This analysis predicts amino acid sites that may be involved in between-clade functional divergence against the background of neutral evolution (Gu 2006). It first estimates a type II functional divergence coefficient (*θ*_II_) that indicates site-specific evolutionary shifts in amino acid biochemical state between clades (i.e., positively charged amino acid on a given site in clade 1 and negatively charged amino acid on the same site in clade 2). Subsequently, it computes the posterior probability that each individual site contributes to the clade-specific functional diversification. We found that both α9 and α10 subunits presented *θ*_II_ values significantly >0 (supplementary table S3, Supplementary Material online), indicating that both subunits may have accumulated a high number of sites showing potential functionally significant amino acid changes when comparing the mammalian versus sauropsid clades (fig. 2*B*, red bars and supplementary table S3, Supplementary Material online). Of note, this analysis identified, in α9 subunits, the aspartate/asparagine (mammals/sauropsid) substitution in the extracellular domain and the alanine/aspartate (mammals/sauropsid) substitution at the exit of the channel pore (fig. 2*B*, asterisks) that we previously reported to be involved in functional differences in calcium permeability between mammalian and avian receptors (Lipovsek et al. 2014). In contrast, neuronal subunits failed to show between-clade functional divergence at the sequence level (fig. 2*B* and supplementary table S3, Supplementary Material online). Overall, the extended molecular evolution analysis of nAChR subunits showed that while neuronal subunits were highly conserved across all vertebrate clades, α9 and α10 hair cell subunits showed a greater degree of between-clade sequence divergence that differentiates mammalian and sauropsid subunits.

**FIg. 3.**
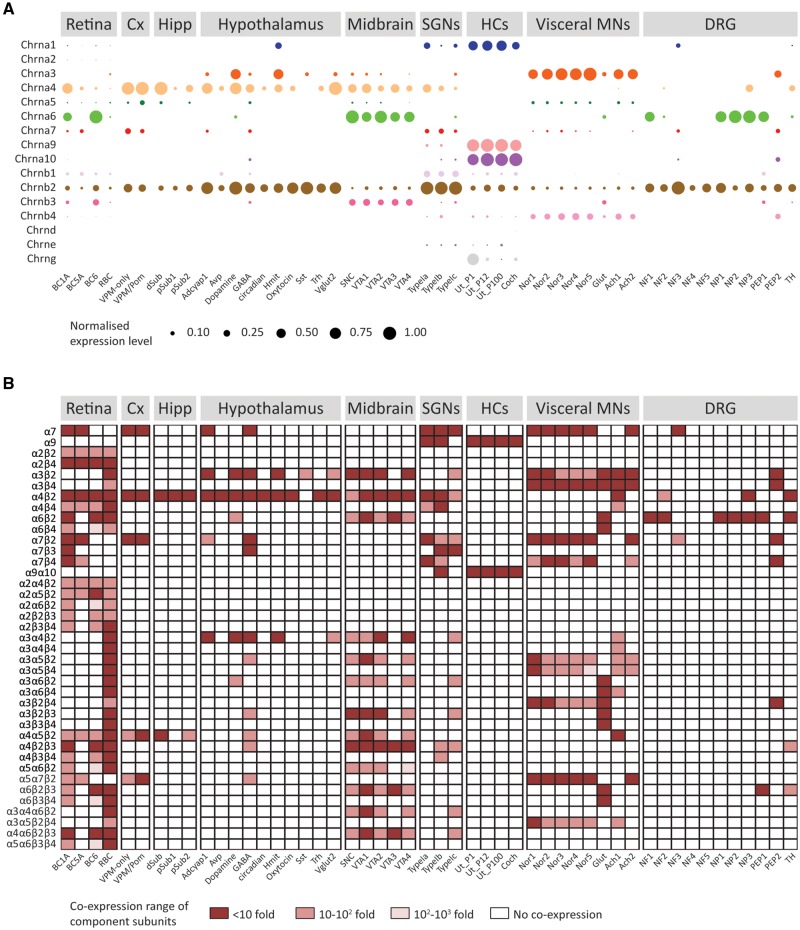
Hair cell nAChR subunits are coexpressed in inner ear hair cells, whereas neuronal subunits show widespread and variable coexpression patterns. (*A*) Normalized mean expression level for nAChR subunits across mouse neuronal and sensory cell types. Circle sizes indicate the mean expression level for each cell type, normalized to the highest value observed within each data set. For detailed explanations of individual cell types refer to main text, Materials and Methods section or the original publications. (*B*) Coexpression of subunits comprising known nAChR assemblies. Dark red squares, all component subunits are coexpressed within a 10-fold range of expression level. Light red squares, all component subunits are coexpressed within a 100-fold range of expression level. Pink squares, all component subunits are expressed within a 1,000-fold range of expression level. White squares, at least one subunit of that receptor assembly in not expressed in that cell type.

The capability to coassemble into functional receptors and the coexpression of nAChR subunits within a given cell delineate the complement of receptors that shape its nicotinic ACh response. Numerous heterologous expression experiments and subunit-specific pharmacological studies have outlined a comprehensive repertoire of functionally validated pentameric assemblies (supplementary table S6, Supplementary Material online, and references therein). However, no systematic gene expression analysis that explores the potential spectrum of nAChRs in a given cell type has been performed. In order to evaluate coexpression patterns, we performed a meta-analysis of gene expression data from ten recent mouse single-cell transcriptomic studies (supplementary table S4, Supplementary Material online). Starting with the gene expression matrices and the cell types identified by each study, we used a Bayesian approach (Kharchenko et al. 2014) to estimate the likelihood of a gene being expressed at any given average level in a given cell type (fig. 3*A* and supplementary table S5 and fig. S3, Supplementary Material online). We next combined these data with the catalog of validated nAChR pentamers (supplementary table S6, Supplementary Material online) and outlined the potential complement of pentameric receptors present in each cell type, by identifying the subunit combinations that are present within a 10-fold, 100-fold, or 1,000-fold range of expression level or altogether absent (fig. 3*B*). As expected, neurons express a range of neuronal nAChR variants with major neuronal types identified by their well characterized nAChRs. For example, visceral motor neurons from thoracic sympathetic ganglia express receptors containing α3 and β4 subunits, whereas cortical neurons express receptors containing α4, β2, and α7 subunits. Ventral midbrain dopaminergic neurons express high levels of the α6 subunit, together with β2 and β3 subunits and variable levels of α3, α4, and α5 subunits. Receptors containing the α2 subunit are observed in different types of retinal neurons (fig. 3). Also, GABAergic, glutamatergic, and dopaminergic neurons from hypothalamus, ventral midbrain, and/or sympathetic ganglia present noticeably different potential complements of nAChRs (fig. 3*B*). Differences in putative nAChRs composition can be observed even between closely related cell types. For example, two subtypes of cortical pyramidal neurons that differ on their projection targets (Chevée et al. 2018) show a significant difference in the expression level of the α5 subunit (Supplementary fig. S3*A*, Supplementary Material online), indicating they could differ on the ratio of α4β2/α4α5β2 receptors on the plasma membrane. Finally, only inner ear hair cells coexpress high levels of α9 and α10 subunits (fig. 3 and supplementary fig. S3*B*, Supplementary Material online).

Taken together the analysis of amino acid sequences and coexpression patterns indicates that, although the α9 and α10 subunits have a restricted expression pattern, they also have the highest clade-specific sequence divergence. On the contrary, neuronal nAChRs present higher sequence conservation together with a widespread expression pattern.

### Divergence of Biophysical Properties Differentiates Neuronal from Hair Cell nAChRs

The restricted coexpression pattern of hair cell α9 and α10 nAChR subunits (fig. 3) and their exclusive coassembly with each other (Elgoyhen et al. 2001; Sgard et al. 2002; Scheffer et al. 2007), together with their higher level of between-clade sequence divergence (fig. 2 and supplementary table S3, Supplementary Material online), indicate the potential for functional innovations across clades through coding sequence changes. In contrast, the high conservation of neuronal subunits (fig. 2 and supplementary table S3, Supplementary Material online) may have resulted in highly conserved functional properties of neuronal receptors assembled from the same subunits. We experimentally tested this prediction by performing a comprehensive side-by-side comparison of the functional properties of the two main neuronal, α4β2 and α7 nAChRs, and the hair cell α9α10 nAChR from three representative species of tetrapod clades. To this end, we injected *Xenopus laevis* oocytes with the corresponding cRNAs and characterized the biophysical properties of ACh responses.

**FIg. 4.**
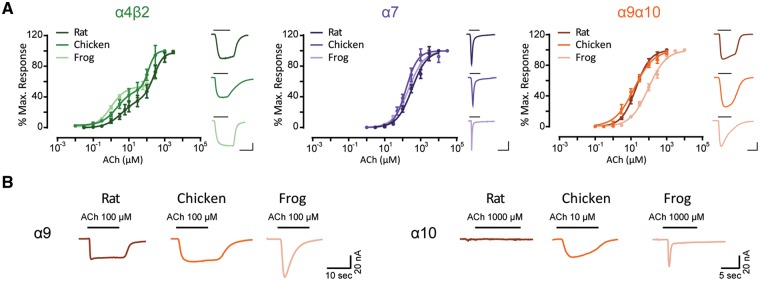
Hair cell nAChRs show differences in ACh apparent affinity, whereas neuronal nAChRs have similar ACh sensitivity. (*A*) Concentration–response curves for neuronal α4β2 and α7 nAChRs and hair cell α9α10 nAChRs from three tetrapod species. Values are mean ± S.E.M. Lines are best fit to the Hill equation (*n* = 4–9). Representative responses evoked by 10 μM (α4β2, rat and chick α9α10) or 100 μM (α7, frog α9α10) ACh are shown next to their respective plots. Scale bars—α4β2: 100 nA, 10 s; α7: 50 nA, 5 s; α9α10: 50 nA, 10 s. (*B*) Representative responses evoked by ACh in oocytes injected with rat, chicken or frog homomeric α9 and α10 subunits (*n* = 2–20).

Oocytes injected with rat (*Rattus norvegicus*), chicken (*Gallus gallus*), or frog (*Xenopus tropicalis*) α4 and β2 cRNAs responded to ACh and showed the characteristic two-component ACh dose–response curves that correspond to the well described (α4)_3_(β2)_2_ and (α4)_2_(β2)_3_ prevalent stoichiometries (fig. 4*A*, table 1, and Moroni et al. [2006]). Oocytes injected with rat, chicken, or frog α7 cRNA responded to ACh with similar apparent affinities (fig. 4*A* and table 1). Finally, rat, chicken, and frog α9 and α10 subunits formed functional heteromeric α9α10 nAChRs that responded to ACh in a concentration-dependent manner (fig. 4*A* and Elgoyhen et al. [2001] and Lipovsek et al. [2012]). The frog α9α10 receptor exhibited a significantly higher apparent affinity than its amniote counterparts (*P* = 0.0026 [vs rat], *P* = 0.0060 [vs chick]—fig. 4*A* and table 1).

**Table 1.** Biophysical Properties and Statistical Comparisons from Rat, Chicken, and Frog α4β2, α7, and α9α10 Receptors.

Species		Rat		Chicken		Frog	
Receptor α4β2							
ACh sensitivity	Mean ± S.E.M. (*n*)	3.11 ± 2.11 (3)	159.76 ± 35.67 (4)	1.07 ± 0.15 (6)	66.00 ± 9.76 (4)	0.75 ± 0.02 (7)	143.71 ± 51.89 (5)
	ANOVA (*P* value)	EC50_1: rat vs chick = 0.1346, rat vs frog = 0.1018, and chick vs frog = 0.6927^**P**^					
		EC50_2: rat vs chick = 0.3743, rat vs frog = 0.7809, and chick vs frog = 0.3743^**P**^					
Desensitization	Mean ± S.E.M. (*n*)	77.13 ± 1.21 (4)		77.68 ± 5.16 (7)		72.70 ± 3.04 (10)	
	ANOVA (*P* value)	Rat vs chick = 0.9336, rat vs frog = 0.7326, and chick vs frog = 0.7204^**P**^					
Ca^2+^ modulation	Mean ± S.E.M. (*n*)	0.36 ± 0.04 (5)		0.55 ± 0.03 (9)		0.60 ± 0.08 (5)	
	ANOVA (*P* value)	Rat vs chick > 0.999, rat vs frog > 0.9999, and chick vs frog > 0.9999^**P**^					
Ca^2+^ permeability	Mean ± S.E.M. (*n*)	72.07 ± 9.62 (4)		78.75 ± 5.04 (6)		81.51 ± 7.91 (6)	
	ANOVA (*P* value)	Rat vs chick = 0.7999, rat vs frog = 0.7892, and chick vs frog = 0.7999^**P**^					
Rectification profile	Mean ± S.E.M. (*n*)	0.07 ± 0.03 (5)		0.03 ± 0.03 (6)		0.02 ± 0.03 (6)	
	ANOVA (*P* value)	Rat vs chick = 0.6838, rat vs frog = 0.4995, and chick vs frog = 0.9437^**P**^					
**Receptor α7**							
ACh sensitivity	Mean ± S.E.M. (*n*)	432.1 ± 123.45 (8)		267.56 ± 87.98 (8)		239.27 ± 33.90 (7)	
	ANOVA (*P* value)	Rat vs chick = 0.365, rat vs frog = 0.6723, and chick vs frog > 0.9999^**NP**^					
Desensitization	Mean ± S.E.M. (*n*)	3.23 ± 0.79 (6)		3.18 ± 0.54 (9)		2.00 ± 0.41 (6)	
	ANOVA (*P* value)	Rat vs chick > 0.9999, rat vs frog = 0.3743, and chick vs frog = 0.2496^**NP**^					
Ca^2+^ modulation	Mean ± S.E.M. (*n*)	0.63 ± 0.10 (4)		0.46 ± 0.06 (4)		0.63 ± 0.08 (4)	
	ANOVA (*P* value)	Rat vs chick > 0.999, rat vs frog > 0.9999, and chick vs frog > 0.9999^**P**^					
Ca^2+^ permeability	Mean ± S.E.M. (*n*)	29.07 ± 7.68 (4)		37.10 ± 11.82 (4)		38.47 ± 3.98 (7)	
	ANOVA (*P* value)	Rat vs chick = 0.7447, rat vs frog = 0.7447, and chick vs frog = 0.8931^**P**^					
Rectification profile	Mean ± S.E.M. (*n*)	0.04 ± 0.02 (5)		0.02 ± 0.003 (5)		0.03 ± 0.01 (5)	
	ANOVA (*P* value)	Rat vs chick = 0.5909, rat vs frog = 0.9706, and chick vs frog = 0.7295^**P**^					
**Receptor α9α10**							
ACh sensitivity	Mean ± S.E.M. (*n*)	19.39 ± 2.08 (9)		17.62 ± 3.33 (6)		110.89 ± 25.00 (7)	
	ANOVA (*P* value)	Rat vs chick > 0.9999, rat vs frog = 0.0026 and chick vs frog = 0.006^**NP**^					
Desensitization	Mean ± S.E.M. (*n*)	64.46 ± 3.65 (5)		60.84 ± 4.23 (9		14.53 ± 3.01 (7)	
	ANOVA (*P* value)	Rat vs chick > 0.9999, rat vs frog = 0.0051 and chick vs frog = 0.0042**^NP^					
Ca^2+^ modulation	Mean ± S.E.M. (*n*)	3.76 ± 0.73 (5)		1.00 ± 0.07 (4)		0.63 ± 0.05 (12)	
	ANOVA (*P* value)	Rat vs chick < 0.0001****, rat vs frog < 0.0001****, and chick vs frog > 0.9981^**P**^					
Ca^2+^ permeability	Mean ± S.E.M. (*n*)	24.89 ± 2.81 (6)		100.28 ± 14.02 (6)		19.56 ± 3.38 (10)	
	ANOVA (*P* value)	Rat vs chick = 0.0299*, rat vs frog > 0.9999, and chick vs frog = 0.0013**^NP^					
Rectification profile	Mean ± S.E.M. (*n*)	1.21 ± 0.07 (11)		2.31 ± 0.34 (10)		0.21 ± 0.05 (8)	
	ANOVA (*P* value)	Rat vs chick = 0.0229*, rat vs frog = 0.0406*, and chick vs frog < 0.0001****^NP^					

NOte.—Values shown are mean ± S.E.M. (*n*). ^P^Parametric and ^NP^nonparametric analysis. ACh sensitivity: EC_50_ value; desensitization rate: percentage current remaining 20 s (5 s for α7 receptors) after peak response to a 10-fold concentration of EC_50_ ACh; Ca^2+^ modulation: current elicited by EC_50_ ACh at Ca^2+^ 0.5 mM relative to Ca^2+^ 3 mM; Ca^2+^ permeability: percentage of remaining current after BAPTA-AM treatment; rectification profile: current recorded at +40 mV relative to that recorded at −90 mV. *P<0.05; **P<0.01; ***P<0.005; ****P<0.0001.

While the α4 and β2 subunits participate exclusively in heteropentameric receptor assemblies, rat α9 (fig. 4*B* and Elgoyhen et al. [1994]), chicken α9 (fig. 4*B* and Lipovsek et al. [2014]), and frog α9 (fig. 4*B*) subunits assembled into functional homomeric receptors. In contrast, rat α10 subunits cannot form functional receptors on their own (fig. 4*B* and Elgoyhen et al. [2001]). Surprisingly, both chicken α10 (fig. 4*B* and Lipovsek et al. [2014]) and frog α10 (fig. 4*B*) subunits assembled into functional homomeric receptors.

**FIg. 5.**
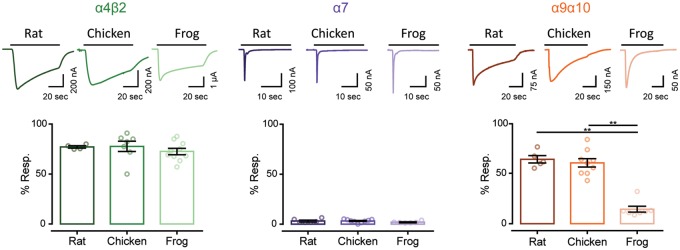
Hair cell nAChRs differ in their desensitization patterns, whereas neuronal receptors show similar profiles. *Top panels*. Representative responses of α4β2, α7, and α9α10 nAChRs to a 60 s (for α4β2 and α9α10) or 30 s (for α7) application of 100 μM ACh for all α4β2 and amniotes α9α10, and 1 mM ACh for all α7 and frog α9α10 nAChRs. *Bottom panels*. Percentage of current remaining 20 s (α9α10 and α4β2) or 5 s (α7) after the peak response, relative to the maximum current amplitude elicited by ACh. Bars represent mean ± S.E.M., open circles represent individual oocytes (*n* = 4–10). **P <* *0.01, one-way ANOVA followed by Dunn’s test (α4β2 nAChRs) or Kruskal–Wallis followed by Holm Sidak’s test (α7 and α9α10 nAChRs).

A defining feature of nAChRs is their desensitization upon prolonged exposure to the agonist (Quick and Lester 2002). Rat and chicken neuronal α4β2 receptors are characterized by slow desensitization kinetics (Lipovsek et al. 2012; Mazzaferro et al. 2017). As shown in figure 5, 70–80% of the maximum current amplitude remained 20 s after the peak response to 100 μM ACh. Similarly, frog α4β2 receptors depicted slow desensitization profiles, with no significant differences when compared with that of rat (*P* = 0.7326) and chicken α4β2 (*P* = 0.7204) nAChRs (table 1 and fig. 5). The frog α7 nAChRs showed fast desensitization with 2–3% of current remaining 5 s after the peak response to 1 mM ACh, similar to that of rat α7 (*P* = 0.3743) and chicken α7 (*P* = 0.2496) nAChRs (table 1 and fig. 5).

The conserved desensitization profiles observed for both types of neuronal receptors was in stark contrast with that of α9α10 receptors. Although rat and chicken α9α10 receptors showed similar and somewhat slow desensitization, with 60–65% of current remaining 20 s after the peak response to ACh (*P* = 0.9999), frog α9α10 nAChRs exhibited significantly higher desensitization when compared with rat α9α10 (*P* = 0.0051) and chicken α9α10 (*P* = 0.0042) receptors (fig. 5 and supplementary table S7, Supplementary Material online).

**FIg. 6.**
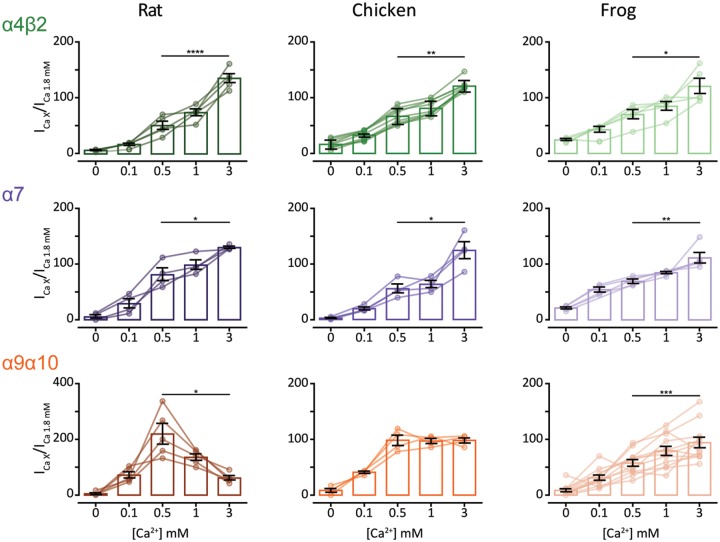
Extracellular Ca^2+^ potentiates neuronal nAChRs but differentially modulates α9α10 nAChRs. ACh response amplitude as a function of extracellular Ca^2+^ concentration. ACh was applied at near-EC50 concentrations (10 μM ACh for all α4β2, rat and chick α9α10 nAChRs and 100 μM ACh for all α7 and frog α9α10 nAChRs). Current amplitudes recorded at different Ca^2+^ concentrations in each oocyte were normalized to the response obtained at 1.8 mM Ca^2+^ in the same oocyte. *V*_h_: −90 mV. Bars represent mean ± S.E.M., open circles represent individual oocytes (*n* = 4–12). *P <* *0.05, **P <* *0.01, ***P <* *0.005, and ****P <* *0.0001, paired *t*-test (rat and frog α4β2 nAChRs and all α7 and α9α10 nAChRs) or Wilcoxon matched pair test (chick α4β2 nAChR)—comparing 0.5 mM Ca^2+^ versus 3 mM Ca^2+^.

Neuronal nAChRs are potentiated by extracellular divalent cations such as Ca^2+^ via an allosteric and voltage-independent mechanism (Mulle et al. 1992; Vernino et al. 1992). The rat α9α10 nAChR, on the other hand, is both potentiated and blocked by physiological concentrations of extracellular divalent cations (Weisstaub et al. 2002). Blockage occurs in the millimolar range, is voltage dependent, and proposed to occur as a result of calcium permeation (Weisstaub et al. 2002). To perform a comparative analysis of calcium modulation on rat, chicken, and frog α4β2, α7, and α9α10 receptors, responses to near-EC_50_ concentrations of ACh were recorded in normal Ringer’s solution at a range of Ca^2+^ concentrations and normalized to the response at 1.8 mM Ca^2+^. For the neuronal α4β2 and α7 receptors from all three species, a similar potentiation pattern was observed, with increasingly higher responses to ACh at greater Ca^2+^ concentrations (fig. 6, top and middle panels). In contrast, responses of α9α10 nAChRs from rat, chicken, and frog exhibited differential modulation by extracellular Ca^2+^. As previously reported for the rat α9α10 receptor, responses to ACh were potentiated and blocked by extracellular Ca^2+^ (fig. 6, bottom left panel and Weisstaub et al. [2002]). The chicken α9α10 receptor also showed peak potentiation of the ACh response at 0.5 mM extracellular Ca^2+^. However, no evident block was observed at higher concentrations of the cation (fig. 6, bottom middle panel). Finally, the frog α9α10 receptor showed potentiation of ACh responses at all Ca^2+^ concentrations assayed, with maximal responses detected at 3 mM Ca^2+^ (fig. 6, bottom right panel).

**FIg. 7.**
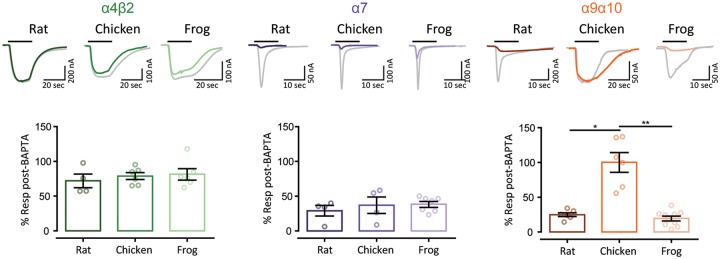
Unlike neuronal nAChRs, α9α10 nAChRs exhibit differential Ca^2+^ contribution to the total inward current. *Top panels*. Representative responses to near-EC_50_ concentration of ACh (10 μM ACh for all α4β2 and amniotes α9α10 nAChRs and 100 μM ACh for all α7 and frog α9α10 nAChRs) in oocytes expressing α4β2, α7, and α9α10 nAChRs, before (gray traces) and after (color traces) a 3-h incubation with BAPTA-AM (*V*_h_ = −70 mV). *Bottom panels*. Percentage of the initial response remaining after BAPTA incubation. Bars represent mean ± S.E.M., open circles represent individual oocytes (*n* = 4–10). ****P <* *0.0001, one-way ANOVA followed by Dunn’s test (α4β2 and α7 nAChRs) or Kruskal–Wallis followed by Holm Sidak’s test (α9α10 nAChRs).

Calcium permeation through nAChRs holds great functional significance for the activation of calcium-dependent conductances and intracellular signaling pathways (Dani and Bertrand 2007). Receptors containing the α7 subunit have high calcium permeability (Séguéla et al. 1993), whereas receptors containing α4β2 subunits have a lower contribution of calcium to the total current (Haghighi and Cooper 2000; Lipovsek et al. 2012). Amniote inner ear hair cell α9α10 nAChRs show differences in the extent of calcium permeability (Lipovsek et al. 2012, 2014). In order to perform a comparative analysis of the extent of the calcium component of ACh responses, we studied the differential activation of the oocyte’s endogenous calcium-activated chloride current (*I*Cl_Ca_). In oocytes expressing a recombinant receptor with high calcium permeability, the *I*Cl_Ca_ is strongly activated upon ACh application (Barish 1983). Incubation of oocytes with the membrane-permeant fast Ca^2+^ chelator BAPTA-AM subsequently abolishes the Cl^−^ component of the total measured current (Gerzanich et al. 1994). Figure 7 shows representative responses to ACh before and after a 3-h incubation with BAPTA-AM for α4β2, α7, and α9α10 nAChRs from rat, chicken, and frog. Although ACh-evoked currents were only slightly affected in all α4β2 nAChRs denoting no major calcium influx (70–80% of current remaining after BAPTA incubation, fig. 7, left panel), all α7 receptors showed a strong reduction of the ACh response after BAPTA incubation (30–40% of current remaining after BAPTA), indicating significant calcium permeation (fig. 7, middle panel). Thus, no interspecies differences in the proportion of calcium current for both the low calcium permeant α4β2 and the highly calcium permeant α7 neuronal receptors were observed (table 1). Conversely, and as previously reported (Lipovsek et al. 2012, 2014), we observed a marked difference in the extent of calcium current between the rat and chicken α9α10 receptors (*P* = 0.0299—fig. 7, right panels and table 1). Moreover, the percentage of remaining response after BAPTA-AM incubation for the frog α9α10 receptor (fig. 7, right panels) was similar to that of the rat receptor (*P* = 0.9999—table 1) and significantly different from that of the chicken α9α10 nAChR (*P* = 0.0013—table 1).

**FIg. 8.**
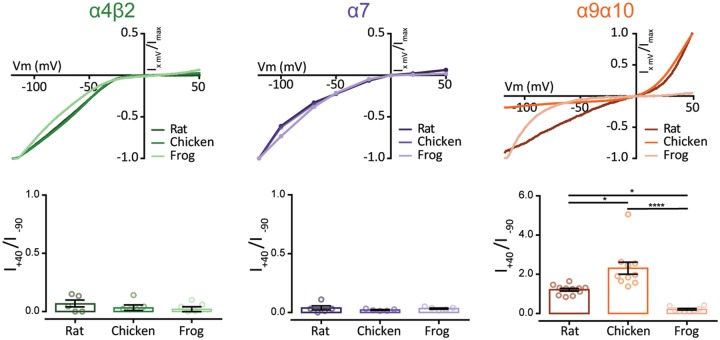
Hair cell, but not neuronal, nAChRs show differential current–voltage relationships across species. *Top panels*. Representative *I*–*V* curves obtained by the application of voltage ramps (−120 to +50mV, 2 s) at the plateau response to 3 μM ACh for α4β2 and α9α10 or by the application of 100 μM ACh at different holding potentials for α7 nAChRs. Values were normalized to the maximal agonist response obtained for each receptor. *Bottom panels*. Ratio of current amplitude at +40 mV relative to −90 mV for each oocyte. Bars represent mean ± S.E.M., open circles represent individual oocytes (*n* = 5–11). *P <* *0.05 and ****P <* *0.0001, one-way ANOVA followed by Dunn’s test (α4β2 and α7 nAChRs) or Kruskal–Wallis followed by Holm Sidak’s test (α9α10 nAChRs).

Neuronal nAChRs are characterized by a strong inward rectification, with negligible current at depolarized potentials (Séguéla et al. 1993; Haghighi and Cooper 2000). This is proposed to be a relevant feature for their roles as presynaptic modulators of neuronal transmission (Haghighi and Cooper 2000). On the other hand, amniote α9α10 nAChRs exhibit a peculiar current–voltage (*I*–*V*) relationship due to a considerable outward current at positive potentials (Elgoyhen et al. 2001; Lipovsek et al. 2012). In order to perform a comparative analysis of the rectification profiles of neuronal and hair cell nAChRs, we obtained *I*–*V* curves and determined the ratio of current elicited at +40 mV to that at −90 mV (*I*_+40_/*I*_−__90_). All neuronal nAChRs exhibited *I*–*V* curves with marked inward rectification with no significant interspecies differences for either α4β2 or α7 tetrapod receptors, presenting *I*_+40_/*I*_−__90_ values below 1 (fig. 8, left and middle panels and table 1). On the contrary, each of the hair cell α9α10 nAChRs analyzed presented a unique *I*–*V* profile. As previously reported, rat α9α10 receptors showed significant outward current at depolarized potentials and greater inward current at hyperpolarized potentials with a *I*_+40_/*I*_−__90_ ratio close to 1 (fig. 8, right panels and Elgoyhen et al. [2001]). The chicken α9α10 receptor showed outward current similar to that of the rat α9α10 receptor, but the inward current was smaller (fig. 8, top right panel), resulting in a significantly higher *I*_+40_/*I*_−__90_ ratio (*P* = 0.0229—table 1). Surprisingly, the frog α9α10 receptor showed an *I*–*V* profile similar to that of neuronal nAChRs, with strong inward rectification, almost no outward current at depolarized potentials (fig. 8, right panels) and a *I*_+40_/*I*_−__90_ below 1, significantly different to that obtained for chick (*P* = 0.0406—table 1) and rat (*P* < 0.0001—supplementary table S9, Supplementary Material online) α9α10 receptors.

### Comparative Functional Analysis of Neuronal and Hair Cell nAChRs Shows Distinct Evolutionary Trajectories

**FIg. 9.**
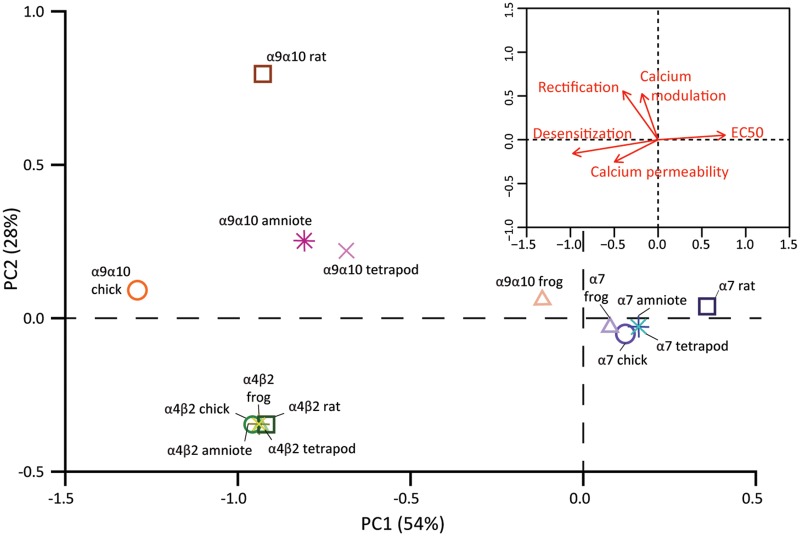
Hair cell nAChRs show great functional divergence, whereas functional properties of neuronal nAChRs are conserved. PCA was conducted using the experimentally determined biophysical properties (table 1). Square symbols represent rat nAChRs, circles represent chick nAChRs, and triangles represent frog nAChRs, α4β2 nAChRs are shown in shades of green, α7 nAChRs in shades of purple, and α9α10 nAChRs in shades of orange. The projected locations of inferred functional states are shown for amniote (stars) and tetrapod (crosses) ancestral receptors and colored in yellow (α4β2), blue (α7), or pink (α9α10). *Inset*. Biplot of the relative contribution of the five biophysical properties to PC1 and PC2.

Altogether, the characterization of individual functional properties of tetrapod nAChRs showed a stark contrast between neuronal and hair cell nAChRs. In order to concomitantly analyze the diversification, or conservation of receptor function, we performed principal component analysis (PCA) on all the functional variables measured on α4β2, α7, and α9α10 receptors from the three species (supplementary tables S8 and S9, Supplementary Material online). The first two principal components (PC) accounted for 82% of the variability (fig. 9). Moreover, the distribution of receptors in PCA space reflected their overall functional differences and similarities. Both neuronal α4β2 and α7 receptors occupied distinct regions, more distant in PC1 than in PC2 denoting that these receptors differ more on ACh apparent affinity, desensitization, and calcium permeability than they do on rectification and calcium modulation (fig. 9, inset). Also, α4β2 and α7 receptors from the different species were located very close together, reflecting the lack of interspecies differences in functional properties. In contrast, the hair cell α9α10 receptors from the different species were distant from each other in PCA space, denoting their extensive functional divergence (fig. 9). Interestingly, the frog α9α10 nAChR was closer to the α7 receptors than to its amniote counterparts, highlighting the overall functional similarity between the amphibian α9α10 and α7 nAChRs.

Amino acid sequence phylogenies, coexpression/coassembly patterns, and functional experiments support the hypothesis of differential evolutionary trajectories for neuronal and hair cell receptors. Further insight could be attained by the evaluation of the receptors present in the last common ancestor of rat and chicken (amniote ancestor) and of rat, chicken, and frog (tetrapod ancestor). To tackle this, we inferred the character state for the functional properties of the α4β2, α7, and α9α10 ancestral receptors (see Materials and Methods). We then projected this predicted functional states onto the functional PCA space of the extant receptors (fig. 9). As expected, the predicted ancestral α4β2 and α7 amniote and tetrapod receptors were located close to their extant counterparts. In contrast, the predicted ancestral states of the α9α10 receptors were localized halfway between the frog α9α10 and the amniote chicken and rat α9α10 extant receptors, reflecting the greater evolutionary distance covered in functional space by the hair cell receptors. In summary, the data portrayed in figure 9 describes a plausible scenario for the functional evolutionary trajectories undertaken by two neuronal and the hair cell nAChRs within the tetrapod lineage.

## Discussion

The expansion of ion channel families on the vertebrate stem branch was mostly driven by two rounds of whole genome duplications (Roux et al. 2017). This has played a crucial role in the evolution of nervous systems and provided raw material that enabled the diversification of cell types (Liebeskind et al. 2015) resulting in the complexity reached by vertebrate brains (e.g., Sugino et al. 2019). Among the different ion channel families, *Cys*-loop receptors are within those that underwent the greatest expansion (Liebeskind et al. 2015). The entire extant complement of nAChRs, which includes 17 different subunits (α1–α10, β1–β4, δ, ε, and γ), was already present in the last common ancestor of all vertebrates (Pedersen et al. 2019). With the exception of some fish species that acquired α11, β1.2, and β5 subunits, for which no expression or functional data have yet been reported (Pedersen et al. 2019), and the loss of the α7-like α8 subunits in mammals, the complement of original vertebrate nAChR subunits has been remarkably conserved. Moreover, nAChRs are unique in that subgroups of the family have distinct roles in synaptic transmission, either in the nervous system, inner ear hair cells, or the neuromuscular junction (Mishina et al. 1986; Dani and Bertrand 2007; Elgoyhen and Katz 2012). In this work, we performed in-depth analyses of coding sequence molecular evolution, subunit coexpression patterns, and comparative functional properties of neuronal and hair cell receptors to explore the potential impact of these segregation of nAChRs subgroups on their respective evolutionary histories. We found that neuronal subunits showed high degree of coding sequence conservation, coupled to greater coexpression variability and conservation of functional properties across tetrapod clades. In contrast, the hair cell α9α10 nAChR showed greater sequence divergence, a highly restricted coexpression pattern and a great degree of variability of functional properties across species. These results indicate that along the tetrapod lineage, neuronal and hair cell nAChRs underwent alternative evolutionary trajectories.

### Functional Conservation of Neuronal nAChRs

The observation that the biophysical functional properties of neuronal α4β2 and α7 nAChRs were conserved in the three tetrapod species analyzed relates to their high degree of amino acid sequence conservation (figs. 1 and 2 and supplementary tables S2 and S3, Supplementary Material online). Cholinergic innervation is pervasive, with almost every area of the brain being influenced by nicotinic signaling (Dani and Bertrand 2007). Moreover, the expression of neuronal nAChR subunits is widespread in the central and peripheral nervous system (fig. 3), where they assemble in multiple combinations (supplementary table S6, Supplementary Material online). Thus, randomly acquired changes in the coding sequence of a given subunit might be deleterious for receptor function in a multitude of heteropentameric assemblies present in diverse neuronal types. Such changes would therefore be under strong negative selection pressure. This is in agreement with the low degree of divergence observed for neuronal subunits and the absence of signatures of positive selection in the protein-coding sequences of α4, β2, and α7 subunits (Lipovsek et al. 2012) and other brain expressed genes (Haygood et al. 2010). However, neurons could alternatively resort to reshuffling of the complement of nAChR subunits being expressed to attain functional divergence. Therefore, and as for most brain expressed genes (Hoekstra and Coyne 2007; Haygood et al. 2010), random changes in noncoding regions that lead to differential expression patterns across brain areas or species may have played a substantial role in delineating the evolutionary trajectories of neuronal nAChRs. Hitherto undescribed evolutionary processes of a higher complexity level that involve changes in the expression pattern, or function, of chaperon proteins that influence post-translational assembly and surface expression of neuronal nAChR subunits (Koperniak et al. 2013; Gu et al. 2016, 2019; Matta et al. 2017; Dawe et al. 2019) may provide an additional substrate for functional divergence.

### Differential Coexpression Patterns of Neuronal and Hair Cell nAChRs

Our meta-analysis of expression patterns across the mouse brain highlights numerous instances of potential functional variability and diversification, even between closely related neuronal types (fig. 3, and supplementary figs. S2 and S3, Supplementary Material online). For instance, cholinergic input controls dopaminergic neuron firing patterns in the midbrain (Mameli-Engvall et al. 2006). Here, coexpression of nAChR subunits greatly differs across the four subtypes of dopaminergic neurons in the ventral tegmental area (VTA) (La Manno et al. 2016 and fig. 3), indicating that they may express differential levels of functionally distinct α4β2 containing (α4β2*) receptors. Inclusion of the α5 subunit can alter α4β2* receptor properties substantially, increasing ACh sensitivity, desensitization kinetics, and Ca^2+^ permeability (Ramirez-Latorre et al. 1996; Tapia et al. 2007; Kuryatov et al. 2008). In addition, incorporation of the β3 subunit to the α4β2* receptor also increases ACh sensitivity, without significantly affecting Ca^2+^ permeability (Tapia et al. 2007; Kuryatov et al. 2008). Moreover, VTA dopaminergic neurons also showed expression of the α6 and α3 subunits, both of which can coassemble with α4, α5 and/or β2 subunits, greatly enhancing the complexity of individual nAChRs-mediated responses of VTA dopaminergic neurons to modulatory cholinergic input. Another interesting example is provided by layer VI cortical pyramidal neurons, whose activity is modulated by a dense cholinergic innervation from the basal forebrain. Here, ACh elicits robust excitatory responses acting on α4β2* nAChRs, with layer VI being one of the few cortical areas that express the accessory α5 nAChR subunit (Proulx et al. 2014). Cortical neurons that project to both the ventral posteromedial nucleus (VPM) and the posteromedial complex of the thalamus express significantly higher levels of the α5 subunit than neurons projecting to the VPM only (supplementary fig. S3*A*, Supplementary Material online). This suggests VPM-only projecting neurons could have a lower density of α4α5β2 compared with α4β2 nAChRs, potentially contributing to differential cholinergic modulation of these subtypes of layer VI neurons, that also show differences in excitability (Landisman and Connors 2007).

Hair cells of the inner ear express high levels of α9 and α10 subunits, along with a number of neuronal (β2 and β4) and muscle (α1, β1, and γ/ε) subunits (fig. 3 and Roux et al. [2016] and Scheffer et al. [2007]). α9α10 nAChRs mediate fast efferent neurotransmission to cochlear and vestibular hair cells in the inner ear (Elgoyhen and Katz 2012) and are characterized by unique pharmacological and biophysical properties (Elgoyhen et al. 1994, 2001; Katz et al. 2000; Weisstaub et al. 2002). Most notably, nicotine, the diagnostic agonist of the nAChRs family, does not act as an agonist of α9α10 receptors, but as a competitive antagonist (Elgoyhen et al. 2001). In inner ear hair cells, no responses to nicotine application are detected (Gomez-Casati et al. 2005; Scheffer et al. 2007), indicating that functional muscle or neuronal nAChRs are not present at the plasma membrane. The presence of neuronal and muscle subunits mRNA may result from redundant or residual transcriptional regulation mechanisms. Moreover, similar “leaky” expression of muscle subunits was detected in a number of neuronal types (fig. 3).

Expression of α9 and α10 subunits is restricted to inner ear hair cells, with a few interesting exceptions. In the inner ear, spiral ganglion neurons (SGNs) provide afferent innervation to cochlear hair cells and express a range of neuronal nAChR subunits (fig. 3 and Hiel et al. [1996] and Morley et al. [1998]). Interestingly, low levels of α9 and α10 subunits are present in SGNs (fig. 3 and Shrestha et al. [2018]) with similarly low coexpression detected by two independent single-cell RNA sequencing studies (Petitpre et al. 2018; Sun et al. 2018). If this low level of α9 and α10 mRNA proves to be more than “transcriptional noise,” then SGNs may be unique among neurons in expressing the hair cell α9α10 receptor. This might be related to the shared developmental origin of SGNs and hair cells at the otic placode (Fritzsch and Beisel 2004; Arendt et al. 2016) and could open the possibility that in addition to neuronal nAChRs, which are thought to partly mediate the nicotinic effect of lateral olivocochlear terminals on afferent dendrites (Reijntjes and Pyott 2016), α9α10 nAChRs may also play a role. Finally, in dorsal root ganglia neurons, α9 expression was not detected, whereas α10 is present at very low levels in only a few subtypes (fig. 3 and Usoskin et al. [2015]). These observations support those reported by quantitative PCR (qPCR) and functional assays (Hone et al. 2012) and provide further evidence that the participation of α9* nAChRs in pain processes is not due to its presence in dorsal root ganglia neurons (Rau et al. 2005; Hone et al. 2012).

Of note, α9 and α10, together with other nAChR subunits, are expressed in other peripheral, nonneuronal, tissues (McIntosh et al. 2009; Zoli et al. 2018). A plausible autocrine/paracrine effect of ACh in these cells can be served by a multiple and redundant battery of nAChRs that might play a signaling function in these peripheral tissues (Zakrzewicz et al. 2017; Criado 2018). Due to the redundancy in pathways for ACh signaling, it is unlikely that the function of α9α10 nAChRs in these peripheral tissues provided the selection forces that shaped the accumulation of nonsynonymous changes on this receptor.

### The α9α10 nAChR and the Evolution of the Efferent Olivocochlear System

The observations that α9 and α10 genes are only coordinately transcribed in inner ear hair cells, together with their ability to only form functional heteromeric receptors when coassembled with each other but not with other nAChR subunits (Elgoyhen et al. 1994, 2001; Scheffer et al. 2007), support our hypothesis that evolutionary changes in the hair cell receptors may have been focused at the coding sequence. Accordingly, vertebrate α9 and α10 subunits exhibit significant sequence divergence (supplementary table S3, Supplementary Material online, and fig. 2), with mammalian α10 subunits showing a higher than expected accumulation of nonsynonymous substitutions that were positively selected (Franchini and Elgoyhen 2006; Lipovsek et al. 2012). In addition, both α9 and α10 subunits show a high number of clade-specific (mammalian vs. sauropsid) functionally relevant amino acid changes (fig. 2, supplementary table S3, Supplementary Material online, and Lipovsek et al. [2014]). Consequently, the biophysical properties of α9α10 receptors drastically changed across tetrapod species (fig. 9 and Lipovsek et al. [2012, 2014]). Since the primary function of the α9α10 receptor is at the postsynaptic side of the olivocochlear synapse, it can be hypothesized that clade-specific differences in efferent modulation of hair cell activity could have shaped the functional properties of α9α10 receptors. Upon the transition to land, the hearing organs of tetrapods underwent parallel evolutionary processes, mainly due to the independent emergence of the tympanic middle ear, at least five times, in separate groups of amniotes (Manley 2017). This was followed by the independent elongation of the auditory sensory epithelia that extended the hearing range to higher frequencies and the elaboration of passive and active sound amplification mechanisms that lead to the fine tuning of sound detection (Dallos 2008; Manley 2017). More importantly, mammals and sauropsids underwent a parallel diversification of hair cell types, segregating, partially in birds but completely in mammals, the phonoreception and sound amplification functions (Koppl 2011). Efferent innervation to hair cells, mediated by α9α10 nAChRs, is an ancestral feature common to all vertebrate species (Sienknecht et al. 2014). In the auditory epithelia, it modulates sound amplification and followed the hair cell diversification pattern: In birds, it mainly targets short hair cells, whereas in mammals, it targets outer hair cells (Koppl 2011). The latter developed a clade-specific sound amplification mechanism driven by the motor protein prestin and termed somatic electromotility (Dallos 2008). Prestin, together with βV giant spectrin, a major component of the outer hair cells’ cortical cytoskeleton which is necessary for electromotility, shows signatures of positive selection in the mammalian clade that may relate to the acquisition of somatic electromotility (Franchini and Elgoyhen 2006; Cortese et al. 2017). Thus, the mammalian clade-specific evolutionary processes observed in both the α9 and α10 subunits (Franchini and Elgoyhen 2006; Lipovsek et al. 2012, 2014) may be related to overall changes in the efferent olivocochlear systems of this clade that is tasked with the modulation of prestin-driven somatic electromotility. A recent high throughput evolutionary analysis identified 167 inner ear expressed genes with signatures of positive selection in the mammalian lineage (Pisciottano et al. 2019). These inner ear genes, including those encoding the α9 and α10 nAChR subunits, can be considered as hotspots for evolutionary innovation in the auditory system across species.

This scenario provides a context for evaluating the relationship between evolutionary trajectories and the functional role of α9α10 receptors. In mammals, the high calcium influx through α9α10 receptors activates large conductance, voltage, and low-calcium-sensitive BK potassium channels mediating hyperpolarization of outer hair cells in higher frequency regions of the cochlea (Wersinger et al. 2010). In contrast, in short hair cells from the chicken basilar papillae, hyperpolarization is served by the ACh-dependent activation of high calcium sensitive SK potassium channels (Fuchs and Murrow 1992; Samaranayake et al. 2004). Moreover, in contrast to adult mammalian hair cells where efferent fibers directly contact outer hair cells, but not the inner hair cells that release glutamate to activate afferent auditory fibers, efferent innervation in birds and amphibians coexists with afferent innervation in the same hair cells. Calcium influx in these hair cells could therefore result in efferent-triggered, ACh-mediated release of glutamate to auditory afferents due to calcium spill over, bypassing sound mechanotransduction. Thus, limiting the extent of calcium influx through α9α10 nAChRs may be paramount to avoid confounding sensory inputs. In this hypothetical scenario, the low calcium permeability of the avian α9α10 nAChR or the very high desensitization kinetics of the amphibian α9α10 nAChR that restrict calcium load could be related to the aforementioned selection pressure.

### Subgroups of nAChRs and Differential Sources of Functional Divergence

Our observations on expression pattern, coding sequence, and functional divergence provide further evidence in support of the proposition that α9 and α10 are not a subtype of brain nicotinic subunit (for review see Morley et al. [2018]), but form a group of their own, characterized by unique expression profile, pharmacological, and biophysical properties (Elgoyhen et al. 1994, 2001; Katz et al. 2000; Weisstaub et al. 2002) and evolutionary history.

The contrasting evolutionary trajectories of neuronal and hair cell receptors, with functional variability stemming from combinatorial coexpression for the former and changes in coding sequence for the latter, support the notion of differential substrates for random change and ensuing functional divergence. For neuronal subunits, the source of random variability may have been rooted on changes in regulatory sequences. In contrast, for the hair cell receptor, random changes in the coding sequence were fixed throughout the evolutionary history of the tetrapod lineage. Interestingly, muscle subunits showed relatively low levels of coding sequence conservation (fig. 2) and, via combinatorial coassembly, muscle cells can toggle between at least two receptor variants (Mishina et al. 1986). This places muscle receptors in between the two extremes of hair cell (isolated) and neuronal (widespread) receptors. A comparative functional study of muscle receptors would further test our hypothesis, with the prediction that a modest level of functional divergence may be encountered, but outweighed by the functional differences between muscle receptor variants.

In summary, the present work provides evidence in support of different evolutionary trajectories for neuronal and hair cell nAChRs. These may have resulted from the differential substrates for random change that dominated evolutionary processes in each receptor subgroup: diversity of coexpression/coassembly patterns for neuronal subunits, changes in coding sequence for hair cell subunits. Finally, the simultaneous analysis of coding sequences, expression patterns, and protein functional properties generated new insights into the evolutionary history of gene paralogs, thus providing further context for the role of nAChRs in neuronal and hair cell synaptic transmission.

## Materials and Methods

All experimental protocols were carried out in accordance with the guides for the care and use of laboratory animals of the National Institutes of Health and the Institutional Animal Care and Use Committee of the Instituto de Investigaciones en Ingeniería Genética y Biología Molecular, “Dr. Héctor N. Torres.”

### Phylogenetic Analysis of Vertebrate nAChRs Subunits

All sequences were downloaded from GenBank (www.ncbi.nlm.nih.gov/genbank; last accessed December 2018), UCSC (http://genome.ucsc.edu/; last accessed December 2018), and Ensembl (www.ensembl.org; last accessed December 2018) databases. The signal peptides of all sequences were excluded from the analysis since they are not present in the mature functional protein. Accession numbers are listed in supplementary table S1, Supplementary Material online. All sequences were visually inspected, and missing and/or incorrect exons were obtained from the NCBI Genome Project traces database (http://blast.ncbi.nlm.nih.gov/Blast.cgi; last accessed December 2018). Sequence alignment was performed using *Clustal*W on the MEGΑ7 software (www.megasoftware.net; last accessed December 2018; Kumar et al. 2016), with the following parameters: for pairwise alignments, gap opening penalty: 10, gap extension penalty: 0.1; for multiple alignments, gap opening penalty: 10, gap extension penalty: 0.2. Protein weight matrix: Gonnet. Residue specific and hydrophilic penalties: ON. Gap separation distance: 4. End gap separation: OFF and no negative matrix was used. The delay divergent cutoff was 30%. The full alignment for the nicotinic subunits from representative vertebrate species is available in supplementary file 1, Supplementary Material online, in fasta format.

The phylogenetic tree of all nAChR subunits was built using the MEGΑ7 software. Positions containing alignment gaps and missing data were eliminated only in pairwise sequence comparisons. The final data set contained a total of 773 positions. The evolutionary distances (i.e., number of amino acid substitutions per site) were computed using the JTT matrix-based method (Jones et al. 1992). The Neighbor-Joining algorithm (Saitou and Nei 1987) was used to generate the initial tree and branch support was obtained by bootstrap test (1,000 replicates) (Felsenstein 1985). The evolutionary history was inferred using the minimum evolution method (Rzhetsky and Nei 1992). A first tree was generated with variation rate among sites modeled by a gamma distribution (fig. 1) and a second tree assuming uniform variation rates among sites (supplementary fig. S1, Supplementary Material online). Overall, tree topology was similar between both methods.

Average percentage sequence identity was calculated for each subunit using the percentage of sequence identity between each pair of sequences (supplementary file 2, Supplementary Material online) from the same category for all sequences and for within or between mammalian and/or sauropsid sequences. For α10 subunits, the average percentage of sequence identity was also calculated for the nonmammalian paraphyletic group. Values obtained are summarized in supplementary table S2, Supplementary Material online.

### Functional Divergence Analysis

The DIVERGE 3.0 software was used to test for functional diversification of nAChR subunits between the mammalian and sauropsid clades. DIVERGE predicts amino acid sites that may be involved in between-clade functional divergence against the background of neutral evolution (Gu 2006). Briefly, it estimates the type II functional divergence coefficient (*θ*_II_) that indicates site-specific evolutionary shifts in amino acid biochemical state between clades and then uses a Bayesian approach to compute the posterior probability that each individual site contributes to the clade-specific functional diversification. Type II sites represent amino acids that are highly conserved within each clade, but in a different biochemically state between clades (i.e., positively charged in clade 1 and negatively charged in clade 2).

To implement the DIVERGE analysis, multiple alignments of protein sequences for each individual subunit were generated using the MEGA7 software as described above. The highly variable amino-terminal signal peptides and intracellular domains were excluded from the analysis. Phylogenetic trees, with a topology corresponding to the species tree, were constructed for each nicotinic subunit by maximum likelihood and the JTT matrix-based method (Jones et al. 1992). The rates among sites were modeled as a gamma distribution. All positions with <95% site coverage were eliminated. α8, β1, and ε nAChR subunits were not included in the analysis due to lack of mammalian and/or sauropsid sequences to perform suitable comparisons. Subsequently, protein sequence alignments and their corresponding phylogenetic trees (supplementary file 3, Supplementary Material online) were used as input data for DIVERGE 3.0 type II functional divergence analysis (Gu et al. 2013), with default parameters. *θ*_II_ and site-specific posterior probabilities were calculated for each subunit. A *θ*_II_ value significantly >0 (*P* < 0.05) indicates that residues conserved within each group have undergone radical changes in amino acid identity between groups (Gu 2006). *z*-Scores were used to test the significant difference of *θ*_II_ coefficients against the null hypothesis (*θ*_II_ = 0) that implies no sites are present in the protein that reflect between-clade functional divergence (supplementary table S3, Supplementary Material online). Site-specific posterior probabilities were computed for all positions along each subunit (supplementary file 3, Supplementary Material online). Sites with posterior probabilities >0.65 for each subunit are highlighted in figure 2*B*.

### Analysis of nAChR Subunit Expression in Single-Cell RNAseq Data Sets

A meta-analysis of single-cell gene expression data from ten studies was performed to describe the expression patterns of nAChR subunits across cell types. Processed gene expression data tables were obtained from ten single-cell RNAseq studies that evaluated gene expression in retina (Shekhar et al. 2016), inner ear sensory epithelium (Burns et al. 2015; McInturff et al. 2018), spiral ganglion (Shrestha et al. 2018), ventral midbrain (La Manno et al. 2016), hippocampus (Cembrowski et al. 2018), cortex (Chevée et al. 2018), hypothalamus (Romanov et al. 2017), visceral motor neurons (Furlan et al. 2016), and dorsal root ganglia (Usoskin et al. 2015). Accession numbers, cell types inferred, and number of cells analyzed are summarized in supplementary table S4, Supplementary Material online. For all data sets, we used the published gene expression quantification and cell type labels. Each data set was analyzed separately. For the retina data set, we used the Smart-Seq2 sequencing data from Vsx2-GFP positive cells (Shekhar et al. 2016). For the gene expression quantification, we only analyzed four cell types that had a minimum number of cells in the data set that allowed reliable fitting to the error models: BC1A, BC5A, BC6, and RBC. From the layer VI somatosensory cortex data set (Cembrowski et al. 2018), we used a subset of the expression matrix that corresponds to day 0 (i.e., control, undisturbed neurons) of their experimental manipulation. For the hypothalamic neurons data set (Romanov et al. 2017), we used a subset that contained only neurons from untreated (control) mice and only quantified gene expression on the ten broad cell types identified. From the ventral midbrain dopaminergic neurons data set (La Manno et al. 2016), we used a subset comprising DAT-Cre/tdTomato positive neurons from P28 mice. For the SGNs data set, we used a subset comprising type I neurons from wild type mice (Shrestha et al. 2018). For the utricle hair cell data sets, we used the normalized expression data of McInturff et al. (2018). For the cochlear hair cell data, we used the normalized expression data from Burns et al. (2015) and continued our analysis with only the ten cochlear hair cells identified. For the visceral motor neurons data set (Furlan et al. 2016), we excluded the neurons that were “unclassified” from further analysis. For the dorsal root ganglia data set (Usoskin et al. 2015), we used a subset containing only successfully classified neurons that were collected at room temperature. Inspection of all data sets for batch effects was performed using the *scater* package (version 1.10.1) (McCarthy et al. 2017). All data analysis was implemented in R (version 3.5.1) and Bioconductor (version 3.8) (http://www.bioconductor.org/; last accessed December 2018), running on RStudio (version 1.1.456) (http://www.rstudio.com/; last accessed December 2018).

The publicly available expression matrices for a number of the data sets contained raw counts (retina, hippocampus, hypothalamus, midbrain, and visceral motor neurons). For each of these data set separately, we performed a normalization step using the *scran* package (version 1.10.2) (Lun et al. 2016) that computes pool-based size factors that are subsequently deconvolved to obtain cell-based size factors.

The normalized expression matrices and cell type information were used as input to quantify cell-type-specific gene expression. Analysis was performed using the *scde* package (version 1.99.1) (Kharchenko et al. 2014). We modeled the gene expression measurements from each individual cell as a weighted mixture of negative binomial and low-magnitude Poisson distributions. The former accounts for the correlated component in which a transcript is detected and quantified, whereas the latter accounts for drop-out events in which a transcript fails to amplify when present. The weighting of the two distributions is determined by the level of gene expression in the cell population (Kharchenko et al. 2014). We then used these error models to estimate the likelihood (joint posterior probability) of a gene being expressed at any given average level in a given cell type (Kharchenko et al. 2014). Probability distributions for all nAChR subunit genes detected in all the cell types analyzed are shown in supplementary figure S2, Supplementary Material online. This whole transcriptome analysis provides accurate estimations of gene expression levels, thus allowing for the comparison of individual genes within a given cell type (i.e., the complement of nAChR subunits) or the analysis of expression level differences between cell types of the same data set (i.e., change in expression level of nAChR subunits between neuronal subtypes). Inferred mean expression values are summarized in supplementary table S5, Supplementary Material online, and normalized mean expression values are depicted in figure 3.

Subsequently, we combined the information about the complement of nAChR subunits for each cell type with a comprehensive catalog of experimentally validated subunit combinations (supplementary table S6, Supplementary Material online, and references therein). We identified the subunit combinations that were present, in each cell type, within a 10-fold, 10- to 100-fold, or 100- to 1,000-fold range of expression level or absent all together (fig. 3*B*). Admittedly, this analysis approach overlooks the complexities of post-translational modifications, receptor assembly, role of chaperone proteins, and transport to the plasma membrane. However, it provides conservative estimates of the maximum potential of combinatorial assembly of subunits and thus a maximum for the repertoire of nAChR assemblies that could be present at the cell membrane.

### Expression of Recombinant Receptors in *X. laevis* Oocytes and Electrophysiological Recordings

Rat and chick α9 and α10 cDNAs subcloned into pSGEM, a modiﬁed pGEMHE vector suitable for *X.* *laevis* oocyte expression studies, were described previously (Elgoyhen et al. 1994, 2001; Lipovsek et al. 2012). Rat α4, β2, and α7 subunit cDNAs subcloned into pBS SK(−) (Agilent Technologies, Santa Clara, CA) were kindly provided by Dr Jim Boulter (University of California, Los Angeles, CA). Chicken α4 and β2 subunit cDNAs subcloned into pCI (Promega, Madison, WI) were kindly provided by Dr Isabel Bermudez-Díaz (Oxford Brookes University, Oxford, UK). Chicken α7 subunit cDNA cloned into pMXT was kindly provided by Dr Jon Lindstrom (University of Pennsylvania) and was then subcloned into pSGEM between *Hin*dIII and *Sal*I sites. Frog α4, β2, α7, α9, and α10 subunits were cloned from whole brain *Xenopus tropicalis* cDNA. Total RNA was prepared from whole brains using the RNAqueous – Micro kit AM1931 (Ambion, Thermo Fisher Scientific, Boston, MA). First strand cDNA synthesis was performed using an oligodT and the ProtoScript Taq RT-PCR kit (New England Biolabs, Ipswich, MA). Second strand synthesis was performed with the NEBNext mRNA Second Strand Synthesis Module kit – E6111S (New England Biolabs). Full length cDNAs for each subunit were PCR amplified (MultiGene 60 OptiMaxTM Thermal Cycler - Labnet International Inc. Edison, NJ) using specific primers (supplementary table S7, Supplementary Material online). PCR products were sequenced and subcloned into pSGEM between *Eco*RI and *Xho*I sites for α9, α7, and β2 nAChRs subunits, between *Hin*dIII and *Xho*I sites for the α10 subunit and between *Eco*RI and *Hin*dIII sites for the α4 subunit. All expression plasmids are readily available upon request.

Capped cRNAs were in vitro transcribed from linearized plasmid DNA templates using the RiboMAX Large Scale RNA Production System-T7 (Promega). The maintenance of *X.* *laevis*, as well as the preparation and cRNA injection of stage V and VI oocytes, has been described in detail elsewhere (Katz et al., 2000). Briefly, oocytes were injected with 50 nl of RNase-free water containing 0.01–1.0 ng of cRNAs (at a 1:1 molar ratio for α9α10 and α4β2 receptors) and maintained in Barth’s solution (in mM): NaCl 88, Ca(NO_3_)_2_ 0.33, CaCl_2_ 0.41, KCl 1, MgSO_4_ 0.82, NaHCO_3_ 2.4, HEPES 10, at 18 °C.

Electrophysiological recordings were performed 2–6 days after cRNA injection under two-electrode voltage clamp with an Oocyte Clamp OC-725B or C amplifier (Warner Instruments Corp., Hamden, CT). Recordings were filtered at a corner frequency of 10 Hz using a 900BT Tunable Active Filter (Frequency Devices Inc., Ottawa, IL). Data acquisition was performed using a Patch Panel PP-50 LAB/1 interface (Warner Instruments Corp.) at a rate of ten points per second. Both voltage and current electrodes were filled with 3 M KCl and had resistances of ∼1 MΩ. Data were analyzed using Clampfit from the pClamp 6.1 software (Molecular Devices, Sunnyvale, CA). During electrophysiological recordings, oocytes were continuously superfused (10 ml min^−1^) with normal frog saline composed of (in mM): 115 NaCl, 2.5 KCl, 1.8 CaCl_2_, and 10 HEPES buffer, pH 7.2. In order to minimize the activation of the oocyte’s native Ca^2+^-sensitive chloride current (*I*Cl_Ca_) by inward Ca^2+^ current through the nAChRs, all experiments, unless otherwise stated, were carried out in oocytes incubated with the membrane-permeant Ca^2+^ chelator 1,2-bis (2-aminophenoxy)ethane-*N*,*N*,*N*′,*N*′-tetraacetic acid-acetoxymethyl ester (BAPTA-AM; 100 μM) for 3 h prior to electrophysiological recordings. This treatment was previously shown to effectively chelate intracellular Ca^2+^ ions and, therefore, to impair the activation of the *I*Cl_Ca_ (Gerzanich et al. 1994). All recordings were performed at −70 mV holding potential, unless otherwise stated.

### Biophysical Properties of nAChRs

Concentration–response curves were obtained by measuring responses to increasing concentrations of ACh. Current amplitudes were normalized to the maximal agonist response in each oocyte. The mean and S.E.M. values of the responses are represented. Agonist concentration–response curves were iteratively fitted, using Prism 6 software (GraphPad Software Inc.), with the equation: *I*/*I*_max_ = *AnH*/(*AnH* + EC_50_*nH*), where *I* is the peak inward current evoked by the agonist at concentration *AnH*, *I*_max_ is current evoked by the concentration of agonist eliciting a maximal response, EC_50_ is the concentration of agonist inducing half-maximal current response, and *nH* is the Hill coefficient.

Desensitization of Ach-evoked currents was evaluated via prolonged agonist applications. The percentage of current remaining 5 s (for α7 nAChRs) or 20 s (for α4β2 and α9α10 nAChRs) after the peak of the response was determined for each oocyte.

The effects of extracellular Ca^2+^ on the ionic currents through nAChRs were studied by measuring the amplitudes of the responses to a near-EC_50_ concentration of ACh (10 μM for all α4β2 and amniote α9α10 nAChRs, and 100 μM for all α7 and frog α9α10 nAChRs) on extracellular Ca^2+^ ranging from nominally 0 to 3 mM at a holding potential of −90 mV (Weisstaub et al. 2002). These experiments were carried out in oocytes injected with 7.5 ng of an oligonucleotide (5′-GCTTTAGTAATTCCCATCCTGCCATGTTTC-3′) antisense to connexinC38 mRNA (Arellano et al. 1995; Ebihara 1996) to minimize the activation of the oocyte’s nonselective inward current through a hemigap junction channel that results from the reduction of external divalent cation concentration. Current amplitudes at each Ca^2+^ concentration were normalized to that obtained in the same oocyte at a 1.8 mM Ca^2+^.

*I*–*V* relationships were obtained by applying 2 s voltage ramps from −120 to +50 mV from a holding potential of −70 mV, at the plateau response to 3 μM ACh. Leakage correction was performed by digital subtraction of the *I*–*V* curve obtained by the same voltage ramp protocol prior to the application of ACh. Generation of voltage protocols and data acquisition were performed using a Patch Panel PP-50 LAB/1 interface (Warner Instruments Corp.) at a rate of ten points per second and the pClamp 7.0 software (Axon Instruments Corp., Union City, CA). Current values were normalized to the maximum amplitude value obtained for each oocyte. The fast desensitizing α7 receptors had negligible plateau currents. For these receptors, responses to 100 μM ACh were obtained at different holding potentials and normalized to the amplitude response at −120 mV in the same oocyte.

Table 1 summarizes the biophysical properties and statistical comparisons from rat, chicken, and frog α4β2, α7, and α9α10 receptors.

### Statistical Analysis

Shapiro–Wilks normality test was conducted using custom routines written in R v3.4.1, through RStudio software v1.0.153. Statistical significance was determined using either parametric paired *t*-test or one-way ANOVA followed by Holm Sidak’s test, or nonparametric Wilcoxon or Kruskal–Wallis tests followed by Dunn’s tests conducted using Prism 6 software (GraphPad Software Inc.). A *P* < 0.05 was considered significant.

### Reagents

All drugs were obtained from Sigma-Aldrich (Buenos Aires, Argentina). ACh chloride was dissolved in distilled water as 100 mM stocks and stored aliquoted at −20 °C. BAPTA-AM was stored at −20 °C as aliquots of a 100 mM stock solution in dimethylsulfoxide, thawed and diluted into Barth’s solution shortly before incubation of the oocytes. ACh solutions in Ringer’s saline were freshly prepared immediately before application.

### PCA of Functional Properties and Inference of Character State of Functional Properties of Ancestral Receptors

PCA was performed on the experimental values obtained for the functional properties of extant nAChRs implementing custom routines written in R v3.4.1 and run in RStudio software v1.0.153. Each of the experimental variables was normalized to the maximum value recorded to allow for equal weighting of the properties (supplementary table S8, Supplementary Material online). The loadings of the empirical variables on the five PCs generated are shown in supplementary table S9, Supplementary Material online, alongside the proportion of the total variability explained by each component. The loadings of each biophysical property on each PC are also shown on the vectors biplot (fig. 9, inset).

### Inference of Character State of Functional Properties of Ancestral Receptors

The pipeline followed to infer the ancestral character state of biophysical properties of nAChRs is schematized on supplementary figure S4, Supplementary Material online. Briefly, we first reconstructed the ancestral tetrapods and amniote DNA sequences of the α4, α7, α9, α10, and β2 nAChRs subunits (supplementary file 4, Supplementary Material online). For that purpose, we used the same ortholog sequences that were used to construct the phylogenetic tree of figure 1, together with a species tree with no branch lengths obtained from Ensembl (https://www.ensembl.org/info/about/speciestree.html; last accessed December 2018). Inferred ancestral DNA sequences for the amniote and tetrapod nodes (supplementary file 4, Supplementary Material online) were obtained, for all three codon positions, by the maximum likelihood method (Nei and Kumar 2000) under the Tamura–Nei model (Tamura and Nei 1993), on the MEGΑ7 software (Kumar et al. 2016). The initial tree corresponds to the provided Species Tree with very strong restriction to branch swap. The rates among sites were treated as a gamma distribution. All positions with <95% site coverage were eliminated.

Subsequently, multiple alignments including extant rat, chick, and frog and ancestral amniote and tetrapod amino acid sequences were performed using MEGΑ7 for each studied nAChR (supplementary file 5, Supplementary Material online). The sequence identity was used to assign branch length values to α7, α4β2, and α9α10 nAChRs trees, corresponding to 1-SeqID (supplementary table S10, Supplementary Material online). Theoretical concatemeric constructions were built for the heteromeric α9α10 nAChRs considering the descripted prevalent (α9)_2_(α10)_3_ stoichiometry (Plazas et al. 2005). For the α4β2 nAChRs, the high sensitivity (α4)_2_(β2)_3_ stoichiometry was used to generate the theoretical concatemeric receptor (supplementary file 5, Supplementary Material online). The resulting trees (supplementary fig. S5, Supplementary Material online) were used, together with the biophysical properties experimentally determined for the extant receptors (table 1) as input data for ancestral character inference. ACh sensitivity (EC_50_ values), desensitization rates (% of remaining *I* 20 s after ACh peak), Ca^2+^ modulation (*I* elicited by ACh at Ca 0.5 mM/Ca 3 mM), Ca^2+^ permeability (% of remaining *I* after BAPTA treatment), and rectification profile (*I*_+40 mV_/*I*_−__90 mV_) for the ancestral amniote and tetrapod receptors were inferred using the *ace* function from the *APE* package v5.2 (Paradis et al. 2004) implemented in R v3.4.1 and RStudio v1.0.153. We used the Brownian motion model (Schluter et al. 1997), where characters evolve following a random walk fitted by maximum likelihood (Felsenstein 1973) for the ancestral character estimations of continuous traits. Reconstructed ancestral states are shown in supplementary figure S6, Supplementary Material online. Finally, using the loadings of the biophysical properties on PC1 and 2 (supplementary table S9, Supplementary Material online, and fig. 9, inset), and the normalized in silico reconstructed biophysical properties inferred for the ancestral receptors we calculated their position on the bidimensional PCA space (fig. 9).

## Supplementary Material

msz290-Supplementary_Data
