# Supplementary material for: Distinct Evolutionary Trajectories of Neuronal and Hair Cell Nicotinic Acetylcholine Receptors
Source: Mol Biol Evol. 2019 Dec 10;37(4):1070–89. doi: 10.1093/molbev/msz290 (PMC7086180; doi:10.1093/molbev/msz290)
Supplement: msz290-Supplementary_Data [file msz290-supplementary_data.zip › msz290-Suppl_Data/Marcovich_et_al_Sup_Material.pdf]

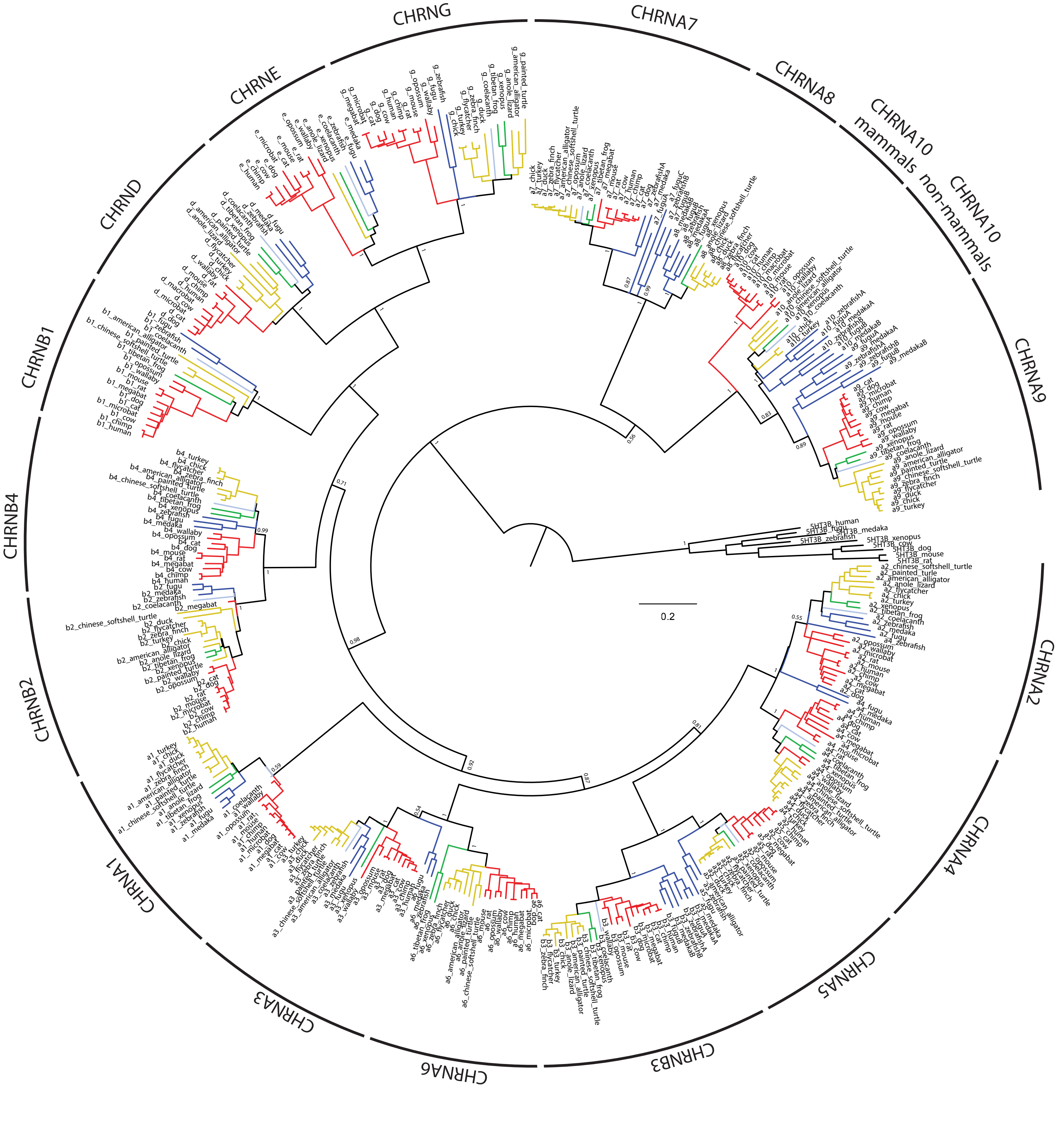

Supplementary Figure 1. Complete minimum evolution phylogenetic tree obtained assuming uniform variation rates among branches. Red branches, mammals; yellow branches, sauropsids; green branches, amphibians; blue branches, fish; light blue branches, coelacanth. The trees were built using minimum evolution method and pairwise deletion for missing sites. The optimal tree with a sum of branch length of 37.49411418 is shown. For clarity, the percentage of replicate trees in which the associated taxa clustered together in the bootstrap test (1,000 replicates) are shown only next to the branches that separate different subunits. The tree is drawn to scale, with branch lengths in the same units as those of the evolutionary distances used to infer the tree.

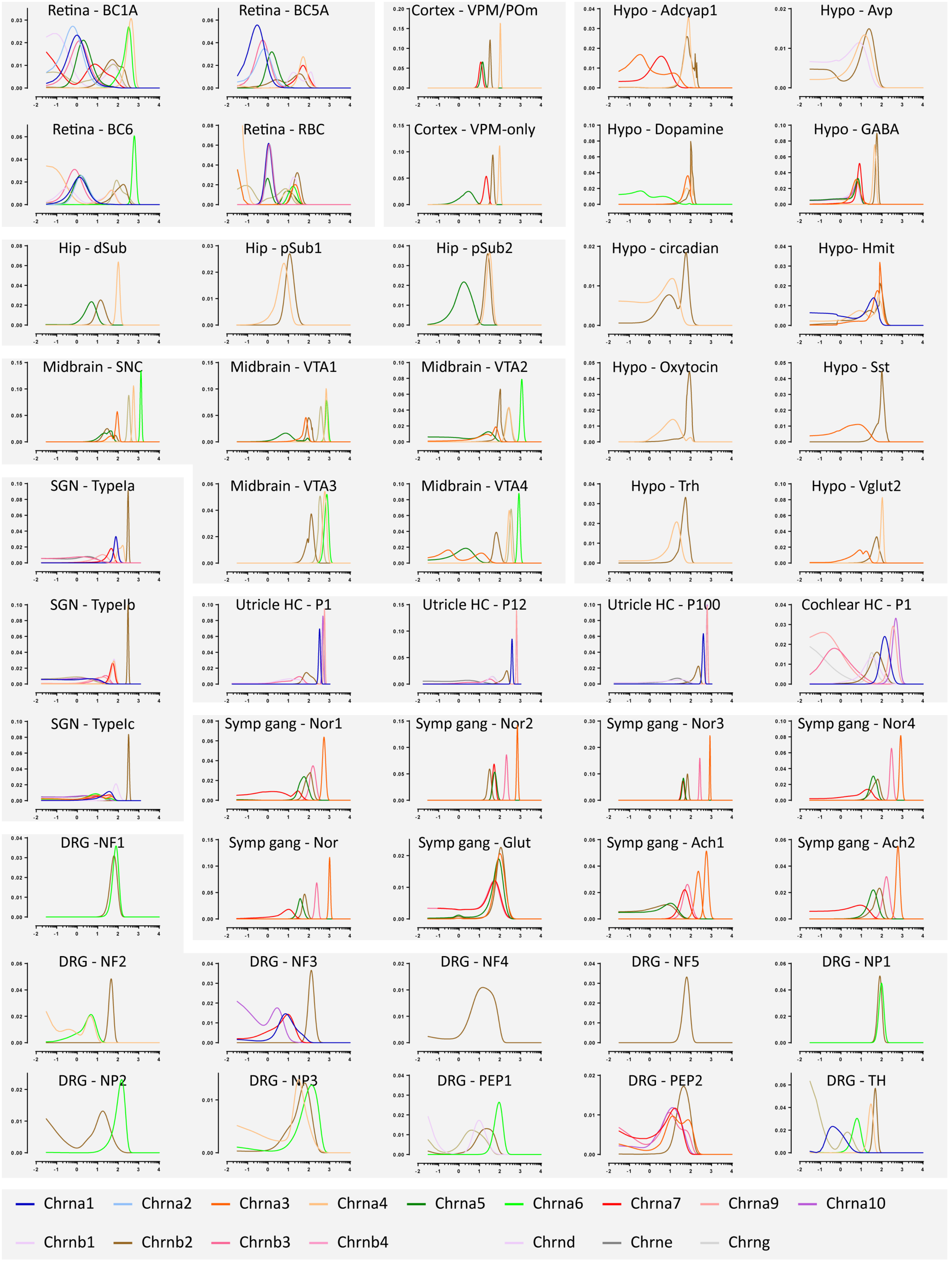

Supplementary Figure 2. Joint posterior probabilities of gene expression levels inferred for the nAChR subunits expressed in the cell types and datasets analysed (see Materials and Methods for details).

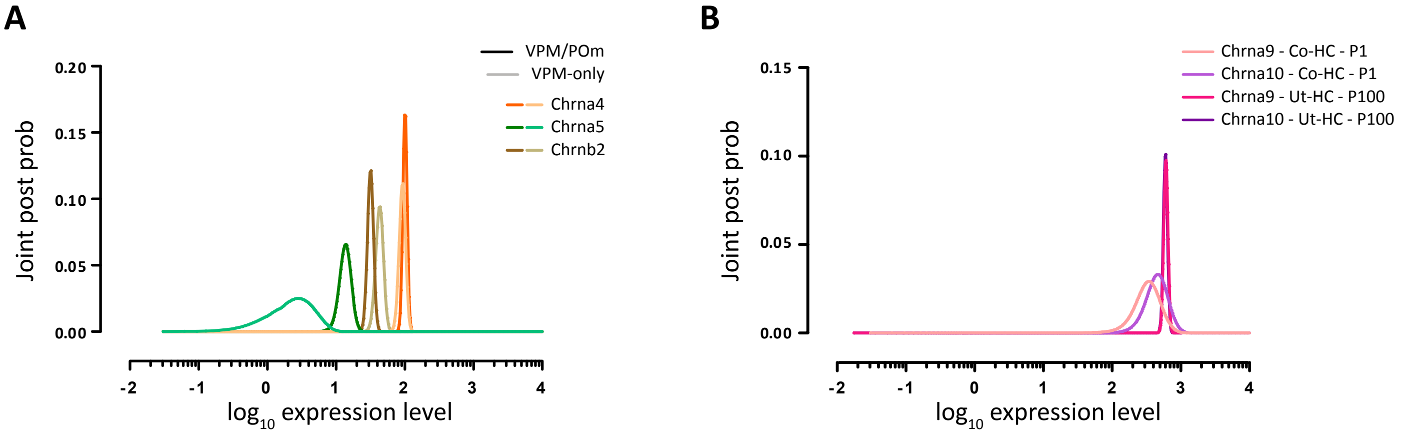

Supplementary Figure 3. A. Estimated joint posterior probability distributions for expression levels of Chrna4, Chrna5 and Chrnb2 in VPM-only and VPM/POm projecting layer VI neurons. **B.** Estimated joint posterior probability distributions for expression levels of Chrna9 and Chrna10 in inner ear cochlear hair cells (P1) and utricle hair cells (P100).

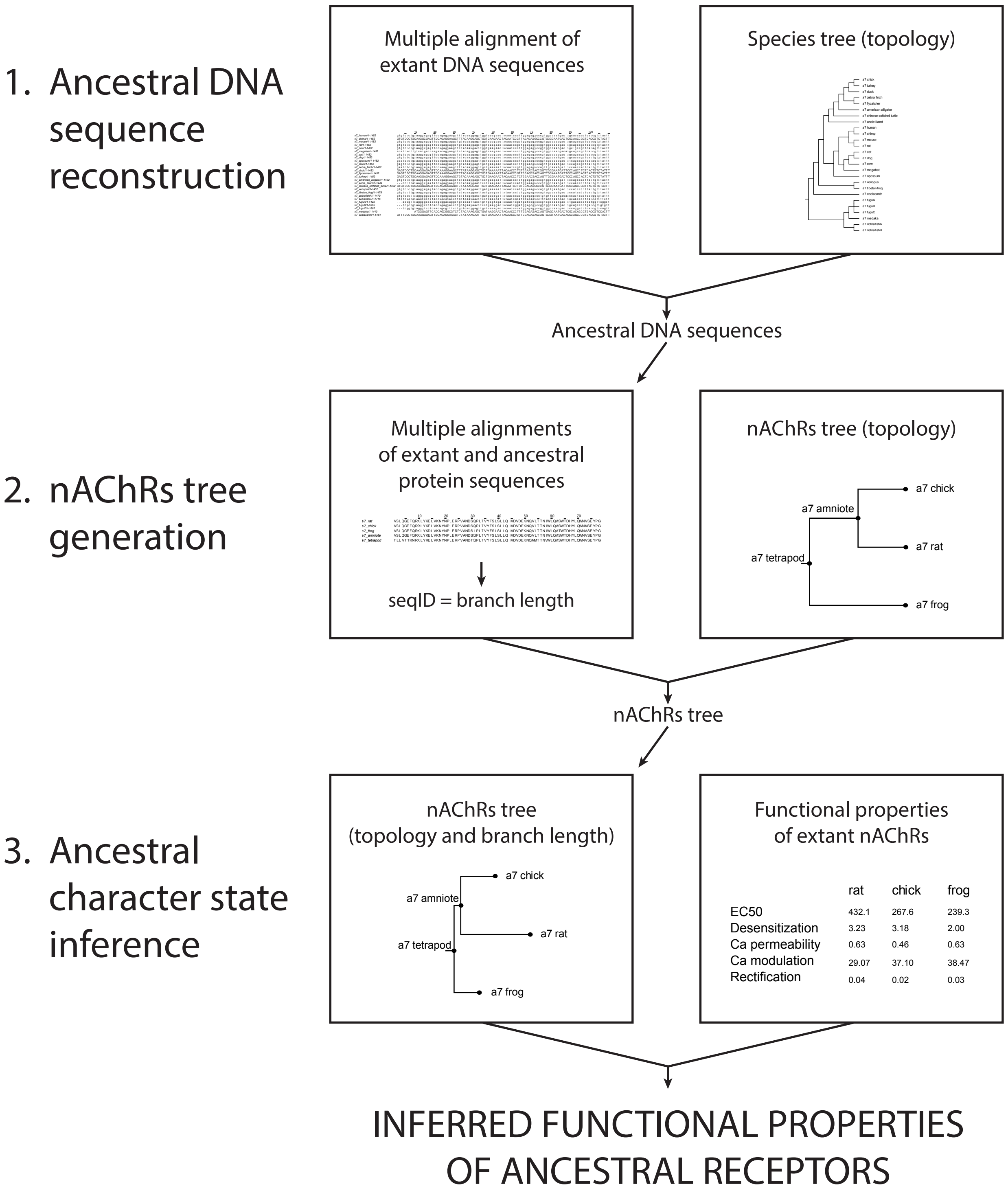

Supplementary Figure 4. Experimental workflow detailing the steps followed for the inference of ancestral character states of the functional properties of tetrapod and amniote ancestral α4β2, α7 and α9α10 receptors.

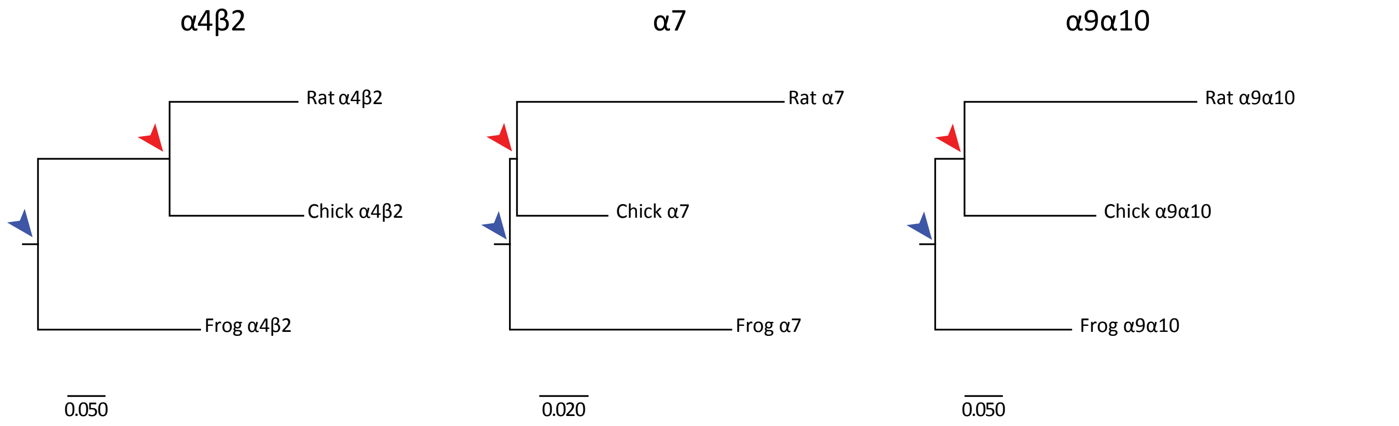

Supplementary Figure 5. nAChRs trees used for the inference of ancestral state states. The tree topology corresponds to the species phylogenetic relationship. The branch lengths correspond to those determined from aminoacid sequence identity analysis between rat-amniote, chick-amniote, amniote-tetrapod and frog-tetrapod pairs of subunits. For the heteromeric α4β2 receptors, branch lengths were calculated assuming a (α4)2(β2)3 assembly. For the heteromeric α9α10 receptors branch lengths were calculated assuming a (α9)2(α10)3 stoichiometry (Plazas et al., 2005). Blue arrows, tetrapod ancestor. Red arrows, amniote ancestor.

Receptor	EC_{50} 1	EC_{50} 2	Desensitization	Ca^{2+} modulation	Ca^{2+} permeability	Rectification
α4β2 frog	0.75	143.71	72.70	0.60	81.51	0.02
α4β2 tetrapod	**1.37**	**130.26**	**74.82**	**0.53**	**78.72**	**0.03**
α4β2 chick	1.07	66.00	77.68	0.55	78.75	0.03
α4β2 amniote	**1.86**	**119.34**	**76.54**	**0.48**	**76.46**	**0.04**
α4β2 rat	3.11	159.76	77.13	0.36	72.07	0.07
α7 frog	239.27		2.00	0.63	38.47	0.03
α7 tetrapod	**291.59**		**2.89**	**0.53**	**35.92**	**0.03**
α7 chick	267.56		3.18	0.46	37.10	0.02
α7 amniote	**293.28**		**2.92**	**0.53**	**35.83**	**0.03**
α7 rat	432.10		3.23	0.63	29.07	0.04
α9α10 frog	110.89		14.53	0.63	19.56	0.21
α9α10 tetrapod	**60.27**		**40.55**	**1.38**	**48.80**	**1.14**
α9α10 chick	17.62		60.84	1.00	100.28	2.31
α9α10 amniote	**49.39**		**46.14**	**1.54**	**55.09**	**1.34**
α9α10 rat	19.39		64.46	3.76	24.89	1.21

Supplementary Figure 6. Inferred ancestral character states for amniote and tetrapod α9α10, α4β2 and α7 nAChRs. The biophysical properties of ancestral receptors inferred by maximum likelihood are shown in black. The values in grey correspond to the biophysical properties of extant receptors determined experimentally (Table 1) and used as input for the inference of ancestral character state. The symbols next to each receptor correspond to the symbols in Figure 8.

Supplementary table S1. Accession numbers for sequences used in the phylogenetic analysis.

	CHRNA1	CHRNA2	CHRNA3	CHRNA4	CHRNA5	CHRNA6	CHRNA7	CHRNA8	CHRNA9	CHRNA10	CHRNB 1	CHRNB 2	CHRNB 3	CHRNB 4	CHRND	CHRNE	CHRNG	5HT3B
**Opossum**	ENSMODG000000094 14	ENSMODG0000 0015940	ENSMODG0000 0011883	ENSMODG0000 0016860	ENSMODG0000 0011857	ENSMODG0000 0010120	ENSMODG0000 0008920	-	ENSMODG0000 0020560	ENSMODG0000 0008246	ENSMODG0000 0006951	ENSMODG0000 0017165	ENSMODG0000 0010082*	ENSMODG0000 0011891	ENSMODG0000 0002646*	ENSMODG0000 0005395	ENSMODG0000 0002666*	
**Wallaby**	ENSMEUG000000036 27*	ENSMEUG00000 004558*	ENSMEUG00000 010338*	ENSMEUG00000 006728*	-	ENSMEUG00000 011281*	ENSMEUG00000 009840*	-	ENSMEUG00000 010143*	ENSMEUG00000 009564*	ENSMEUG00000 008924*	ENSMEUG00000 003725*	ENSMEUG00000 011355*	ENSMEUG00000 001043*	ENSMEUG00000 011484*	ENSMEUG00000 012185*	ENSMEUG00000 011510*	
**Rat**	ENSRNOG000000182 86	ENSRNOG00000 017424	ENSRNOG00000 013829	ENSRNOG00000 011202	ENSRNOG00000 013610	ENSRNOG00000 012283	ENSRNOG00000 010853	-	ENSRNOG00000 002484	ENSRNOG00000 020293	ENSRNOG00000 014698	ENSRNOG00000 020778	ENSRNOG00000 012448	ENSRNOG00000 014427	ENSRNOG00000 019527	ENSRNOG00000 003777	NM_019145.1	
**Mouse**	ENSMUSG000000271 07	ENSMUSG00000 022041	ENSMUSG00000 032303	ENSMUSG00000 027577	ENSMUSG00000 035594	ENSMUSG00000 031491	ENSMUSG00000 030525	-	ENSMUSG00000 029205	ENSMUSG00000 066279	ENSMUSG00000 041189	ENSMUSG00000 027950	ENSMUSG00000 031492	ENSMUSG00000 035200	ENSMUSG00000 026251	ENSMUSG00000 014609	ENSMUSG00000 026253	ENSMUSG00000 008590
**Cat**	ENSFCAG0000001410 3*	ENSFCAG00000 028158	ENSFCAG00000 001932	ENSFCAG00000 030656	ENSFCAG00000 001930	ENSFCAG00000 022358	ENSFCAG00000 036300	-	ENSFCAG00000 035547	ENSFCAG00000 023105	ENSFCAG00000 009612	ENSFCAG00000 022169*	ENSFCAG00000 010080	ENSFCAG00000 001933	ENSFCAG00000 030943	ENSFCAG00000 038719	ENSFCAG00000 005681	ENSFCAG00000 004982
**Dog**	ENSCAFG0000001328 3	ENSCAFG00000 008499	ENSCAFG00000 001710*	ENSCAFG00000 012893	ENSCAFG00000 001745*	ENSCAFG00000 005444	ENSCAFG00000 010220*	-	ENSCAFG00000 015915	ENSCAFG00000 024527	ENSCAFG00000 016315	ENSCAFG00000 017172	ENSCAFG00000 005460	ENSCAFG00000 023904	ENSCAFG00000 011283	ENSCAFG00000 015838	ENSCAFG00000 024807*	ENSCAFG00000 013576
**Cow**	ENSBTAG0000001825 3	ENSBTAG00000 002252	ENSBTAG00000 003130	ENSBTAG00000 017198	ENSBTAG00000 013657*	ENSBTAG00000 014056	ENSBTAG00000 015775	-	ENSBTAG00000 015252	ENSBTAG00000 055237	ENSBTAG00000 019242	ENSBTAG00000 007517	ENSBTAG00000 014050	ENSBTAG00000 003132	ENSBTAG00000 011390*	ENSBTAG00000 004908	ENSBTAG00000 045943	ENSBTAG00000 031330
**Human**	ENSG00000138435	ENSG000001209 03	ENSG000000806 44	ENSG000001012 04	ENSG000001696 84	ENSG000001474 34	ENSG000001753 44	-	ENSG000001743 43	ENSG000001297 49	ENSG000001701 75	ENSG000001607 16	ENSG000001474 32	ENSG000001179 71	ENSG000001359 02	ENSG000001085 56	ENSG000001968 11	ENSG000001493 05
**Chimp**	ENSPTRG0000001265 8	ENSPTRG000000 20105	ENSPTRG000000 07347	ENSPTRG000000 13735	ENSPTRG000000 07346	ENSPTRG000000 20210	ENSPTRG000000 06865	-	ENSPTRG000000 16003	ENSPTRG000000 03199	ENSPTRG000000 08689	ENSPTRG000000 01389	ENSPTRG000000 20209	ENSPTRG000000 07348	ENSPTRG000000 13040	ENSPTRG000000 08612	ENSPTRG000000 22726	
**Megabat**	ENSPVAG0000000416 4	ENSPVAG00000 014424	ENSPVAG00000 009191	ENSPVAG00000 014109	ENSPVAG00000 009186	ENSPVAG00000 015371*	ENSPVAG00000 017796*	-	ENSPVAG00000 003814	ENSPVAG00000 016436	ENSPVAG00000 005067	XM_023534307. 1*	ENSPVAG00000 015373	ENSPVAG00000 009193	ENSPVAG00000 007491	ENSPVAG00000 000127	ENSPVAG00000 007492*	
**Microbat**	ENSMLUG000000156 98	ENSMLUG00000 001512	-	Manual search on traces	-	ENSMLUG00000 005231	ENSMLUG00000 005856	-	ENSMLUG00000 000893	ENSMLUG00000 013586	ENSMLUG00000 009921	ENSMLUG00000 007541	ENSMLUG00000 027322	-	ENSMLUG00000 011213	ENSMLUG00000 015682*	ENSMLUG00000 011237	
**Chick**	ENSGALG0000000930 1	ENSGALG00000 016565	ENSGALG00000 003014	ENSGALG00000 005801	ENSGALG00000 003021	ENSGALG00000 015382	ENSGALG00000 004096	ENSGALG00000 015336	ENSGALG00000 014268	NM_001101036. 1	-	ENSGALG00000 002707	ENSGALG00000 015384	ENSGALG00000 003005	ENSGALG00000 007899	-	ENSGALG00000 007906	
**Turkey**	ENSMGAG000000097 52	ENSMGAG00000 014105	ENSMGAG00000 003578	ENSMGAG00000 006295	ENSMGAG00000 003610*	-	ENSMGAG00000 005132	-	ENSMGAG00000 012228	XM_010706263. 2	-	ENSMGAG0000 0006636*	ENSMGAG0000 0007510	ENSMGAG0000 0003559*	ENSMGAG0000 0008354	-	ENSMGAG0000 0008414	
**Duck**	ENSAPLG0000000780 3*	ENSAPLG000000 09491*	ENSAPLG000000 16121	ENSAPLG000000 07776	ENSAPLG000000 16223*	ENSAPLG000000 12469	ENSAPLG000000 10434	ENSAPLG000000 07724	ENSAPLG000000 13711*	-	-	ENSAPLG000000 09547*	ENSAPLG000000 12846	ENSAPLG000000 16042	-	-	ENSAPLG000000 15469*	
**Flycatcher**	ENSFALG0000000303 4	ENSFALG000000 03731	ENSFALG000000 10658	ENSFALG000000 10418	ENSFALG000000 10653	ENSFALG000000 05209	ENSFALG000000 12362*	ENSFALG000000 01025	ENSFALG000000 02853*	XM_016305564. 1	-	ENSFALG000000 01220	ENSFALG000000 05194	ENSFALG000000 10665	ENSFALG000000 07740	-	ENSFALG000000 07743	
**Zebra finch**	ENSTGUG000000088 27	-	ENSTGUG00000 003469	ENSTGUG00000 007453	ENSTGUG00000 003451*	ENSTGUG00000 000216*	ENSTGUG00000 005850	ENSTGUG00000 000135	ENSTGUG00000 008712	-	-	ENSTGUG00000 004092	ENSTGUG00000 000227	XM_002191803. 3	-	-	ENSTGUG00000 009924	
**Anole lizard**	ENSACAG000000052 93	ENSACAG00000 002388	-	ENSACAG00000 005817	-	ENSACAG00000 005512	ENSACAG00000 008436	ENSACAG00000 005448	ENSACAG00000 011789	NW_003340045 .1	-	ENSACAG00000 015594	ENSACAG00000 005630	-	ENSACAG00000 010984	ENSACAG00000 014790*	ENSACAG00000 010910	
**American alligator**	XM_006267516.3	XM_006274660. 2	XM_006261354. 3	XM_019484414. 1	XM_006261353. 3	XM_006276967. 2	XM_014599894. 2	-	XM_019478038. 1	XM_019480144. 1	XM_006275274. 2	XM_019497730	XM_006276963. 2	XM_014605947	XM_019484776. 1	-	NW_017709597 .1*	
**Chinese softshell turtle**	ENSPSIG0000000334 8	ENSPSIG000000 12597	ENSPSIG000000 16882	ENSPSIG000000 12045	-	ENSPSIG000000 06517	ENSPSIG000000 10525	ENSPSIG000000 09608	ENSPSIG000000 17539	XM_014580618. 2	ENSPSIG000000 09407	ENSPSIG000000 06807*	ENSPSIG000000 17586*	ENSPSIG000000 17214	-	ENSPSIG000000 03627*	ENSPSIG000000 11304*	
**Painted turtle**	ENSCPBG0000000379 8	ENSCPBG00000 022315	ENSCPBG00000 024258	ENSCPBG00000 027193	ENSCPBG00000 024257	ENSCPBG00000 027581*	-	-	ENSPSIG000000 17539*	XM_024109380. 1	ENSCPBG00000 004679	ENSCPBG00000 024641*	ENSCPBG00000 027579	ENSCPBG00000 024259	ENSCPBG00000 016193	ENSCPBG00000 016561*	ENSCPBG00000 016190	
**Xenopus tropicalis**	ENSXETG0000002542 0	ENSXETG000000 17553	ENSXETG000000 31723	ENSXETG000000 23746	ENSXETG000000 23896*	ENSXETG000000 10249*	ENSXETG000000 16395	ENSXETG000000 18003	ENSXETG000000 12803	XM_002942872. 4	-	ENSXETG000000 03846	ENSXETG000000 10250	ENSXETG000000 33968*	ENSXETG000000 27884	ENSXETG000000 14957	ENSXETG000000 13994*	ENSXETG000000 31022
**Tibetan frog**	XM_018572442.1	XM_018570301. 1	-	XM_018560101. 1	-	XM_018557911. 1	XM_018564234. 1	-	XM_018564438. 1	-	XM_018573841. 1	XM_018570082. 1	XM_018557907. 1	XM_018572684. 1	XM_018563344. 1	-	XM_018572139. 1	
**Coelacanth**	ENSLACG0000000010 9*	ENSLACG000000 03345	XM_006001841. 1	ENSLACG000000 10383*	ENSLACG000000 02798	-	ENSLACG000000 05760*	-	ENSLACG000000 09570	ENSLACG000000 05922	ENSLACG000000 08409	ENSLACG000000 15217	ENSLACG000000 02288	ENSLACG000000 04838*	ENSLACG000000 15676	ENSLACG000000 02691	ENSLACG000000 15771	
**Fugu**	XM_003961904.2	ENSTRUG00000 008002*	ENSTRUG00000 004067*	ENSTRUG00000 010213	ENSTRUG00000 005757	ENSTRUT000000 02729.2	ENSTRUG00000 016472	ENSTRUG00000 002223	ENSTRUG00000 010253	ENSTRUG00000 003807	XM_011618734. 1	ENSTRUG00000 005762	ENSTRUG00000 006363	ENSTRUG00000 025609	ENSTRUG00000 007767*	ENSTRUG00000 016224	ENSTRUG00000 014376*	ENSTRUG00000 013084
						ENSTRUT000000 16710.2	NC_018902.1	ENSTRUG00000 005961	ENSTRUG00000 013071	NW_004072435 .1			ENSTRUG00000 001764					
							NC_018896.1											
**Medaka**	XM_020713072.2	XM_011491375. 3	ENSORLG00000 013118	ENSORLG00000 000865*	ENSORLG00000 020077	ENSORLT000150 28636.1	ENSORLG00000 018774	ENSORLG00000 003203	ENSORLG00000 004390	NC_019871.2	-	ENSORLG00000 009700	ENSORLG00000 003226	ENSORLG00000 013114	ENSORLG00000 026994	ENSORLG00000 008035*	-	ENSORLG00000 014619
							ENSORLG00000 030549	ENSORLG00000 014282	ENSORLG00000 005701	XM_011483838. 3			ENSORLG00000 014290					
**Zebrafish**	ENSDARG000000090 21	NM_001040327. 1	ENSDARG00000 100991	NM_001048063. 1	ENSDARG00000 003420	ENSDARG00000 055559	ENSDARG00000 101522	ENSDARG00000 087224	ENSDARG00000 054680	NM_001044804. 1	NM_001253810. 1	ENSDARG00000 017790	ENSDARG00000 052764	ENSDARG00000 101677	ENSDARG00000 019342	ENSDARG00000 034307	ENSDARG00000 086647*	ENSDARG00000 061749
							ENSDARG00000 101702		ENSDARG00000 099181	XM_021481417. 1			ENSDARG00000 038508					

Accession numbers correspond to Ensembl or Genbank databases. * Sequences mended and/or completed using trace fragments obtained from the NCBI Genome Project traces database.

Supplementary table S2. Mean percentage sequence identity (%seqID).

	All	Mammals	Mammals-Sauropsid	Sauropsid
CHRNA1	83.98 (26)	93.40 (11)	85.44	88.98 (9)
CHRNA2	73.99 (24)	81.02 (11)	71.68	82.21 (7)
CHRNA3	80.91 (23)	89.78 (10)	83.50	91.79 (8)
CHRNA4	73.63 (26)	80.77 (11)	74.14	90.06 (9)
CHRNA5	84.55 (21)	90.65 (9)	86.18	96.27 (7)
CHRNA6	77.61 (24)	88.09 (11)	80.31	84.93 (8)
CHRNA7	79.22 (26)	92.97 (9)	89.69	96.97 (8)
CHRNA8	77.63 (12)	-	-	91.49 (6)
CHRNA9	72.16 (29)	90.90 (11)	74.23	88.28 (9)
CHRNA10	64.93 (24)	88.98 (11)	61.29	75.38 (5)
CHRNB1	70.47 (18)	86.24 (11)	63.82	73.20 (3)
CHRNB2	77.16 (26)	88.70 (11)	75.83	76.87 (9)
CHRNB3	81.47 (28)	88.41 (11)	84.59	91.83 (8)
CHRNB4	72.12 (23)	80.83 (10)	71.75	85.65 (7)
CHRND	71.54 (22)	87.85 (10)	68.02	76.59 (6)
CHRNE	67.34 (16)	83.08 (10)	58.60	-
CHRNG	69.12 (24)	86.81 (11)	63.41	79.55 (8)

Values were calculated using pairs of sequences of all vertebrates, mammals or sauropsids nAChRs subunits. (n) Indicates the number of orthologues from each subunit included in the calculations. White background: muscle subunits; light background grey: neuronal subunits; dark grey background: hair cells subunits.

Supplementary table S3. Type II functional divergence coefficients for nAChR subunits

	**θ_{II} ± SE**	**P value**	**N sites**
CHRNA1	0.0082 ± 0.03	0.41	1
CHRNA2	-0.02 ± 0.03	0.23	0
CHRNA3	0.03 ± 0.03	0.19	1
CHRNA4	0.01 ± 0.03	0.33	0
CHRNA5	0.03 ± 0.03	0.12	2
CHRNA6	0.01 ± 0.03	0.41	0
CHRNA7	0.02 ± 0.02	0.16	3
CHRNA9	0.05 ± 0.02	0.038	15
CHRNA10	0.1 ± 0.04	0.0071	10
CHRNB2	0.01 ± 0.02	0.30	0
CHRNB3	0.02 ± 0.03	0.29	1
CHRNB4	0.05 ± 0.03	0.060	1
CHRND	0.03 ± 0.05	0.28	4
CHRNG	0.08 ± 0.05	0.051	3

θ_{II}, coefficient of type II functional divergence. SE, standard error. N sites, number of residues per nicotinic subunit with posterior probabilities for type II functional divergence greater than 0.65.

Supplementary table S4. Single-cell RNA sequencing datasets.

Publication	DOI	Accession	Tissue	mean_genes	Cell_types	#Cells
Shekar, et al. 2016	10.1016/j.cell.2016.07.054	GSE81905	Retina	5372	BC1A	14
					BC5A	24
					BC6	17
					RBC	99
Chevee, et al. 2018	10.1016/j.celrep.2017.12.046	GSE107632	Somatosensory cortex	5614	VPMonly	130
					VPM/Pom	210
Cembrowski, et al. 2018	10.1016/j.cell.2018.03.031	GSE100449	Hippocampus	5218	dSub	72
					pSub1	69
					pSub2	102
Romanov	10.1038/nn.4462	GSE74672	Hypothalamus	3887	Adcyap1	34
					Avp	25
					Dopamine	41
					GABA	311
					circadian	31
					Hmit	24
					Oxytocin	37
					Sst	38
					Trh	55
					Vglut2	137
LaManno, et al. 2016	10.1016/j.cell.2016.09.027	GSE76381	Ventral midbrain	4106	DA_SNC	73
					DA_VTA1	47
					DA_VTA2	28
					DA_VTA3	26
					DA_VTA4	69
Shrestha, et al. 2018	10.1016/j.cell.2018.07.007	GSE114997	Spiral ganglion	8298	SGN_TypeIa	63
					SGN_TypeIb	71
					SGN_TypeIc	45
McInturff	10.1242/bio.038083	GSE115934.	utricle HC	5578	HC_P1	37
					HC_P12	50
					HC_P100	25
Burns	10.1038/ncomms9557	GSE71982	inner ear	7143	Co_HC	10
Furlan, et al. 2016	10.1038/nn.4376	GSE78845	Sympathetic ganglion	6297	Nor1	10
					Nor2	39
					Nor3	103
					Nor4	18
					Nor5	23
					Glu	4
					Ach1	8
					Ach2	8
Usoskin	10.1038/nn.3881	GSE59739	DRG	3821	NF1	21
					NF2	43
					NF3	10
					NF4	16
					NF5	24
					NP1	80
					NP2	19
					NP3	10
					PEP1	50
					PEP2	14
					TH	142

Accession information for the datasets used on the analysis of nAChR subunits co-expression patterns. mean_genes, average number of genes per cell detected in each dataset. Cell_types, final cell types, as inferred in each publication, used to estimate the expression levels of nAChR subunit genes. #Cells, number of cells for each cell type present in the final datasets analysed.

Supplementary table S5. Mean expression of nAChR subunits genes across cell types.

	Retina				Somatosensory cortex		Hippocampus			Hypothalamus									
	BC1A	BC5A	BC6	RBC	VPM-only	VPM/Pom	dSub	pSub1	pSub2	Adcyap1	Avp	Dopamine	GABA	circadian	Hmit	Oxytocin	Sst	Trh	Vglut2
Chrna1	3.46	0.72	3.18	1.24	0.00	0.00	0.00	0.00	0.00	0.00	0.00	0.00	0.00	0.00	24.85	0.00	0.00	0.00	0.00
Chrna2	2.93	1.41	3.17	1.37	0.00	0.00	0.00	0.00	0.00	0.00	0.00	0.00	0.00	0.00	0.00	0.00	0.00	0.00	0.00
Chrna3	0.00	0.00	0.00	13.35	0.00	0.00	0.00	0.00	0.00	8.33	0.00	62.03	5.92	0.00	59.68	0.00	8.54	0.00	9.97
Chrna4	382.03	52.93	25.66	6.74	92.58	100.99	99.88	6.10	30.51	68.76	14.87	80.34	43.55	10.23	37.64	17.36	0.00	17.47	100.82
Chrna5	5.35	2.48	3.18	8.01	3.05	13.80	5.36	0.00	2.68	0.00	0.00	0.00	4.89	0.00	0.00	0.00	0.00	0.00	0.00
Chrna6	267.64	0.00	601.04	10.78	0.00	0.00	0.00	0.00	0.00	0.00	0.00	5.67	0.00	0.00	0.00	0.00	0.00	0.00	0.00
Chrna7	19.29	52.13	0.00	0.00	20.54	11.37	0.00	0.00	0.00	5.39	0.00	0.00	7.56	0.00	0.00	0.00	0.00	0.00	0.00
Chrna9	0.00	0.00	0.00	0.00	0.00	0.00	0.00	0.00	0.00	0.00	0.00	0.00	0.00	0.00	0.00	0.00	0.00	0.00	0.00
Chrna10	0.88	0.00	0.00	0.00	0.00	0.00	0.00	0.00	0.00	0.00	0.00	0.00	4.53	0.00	0.00	0.00	0.00	0.00	0.00
Chrnb1	34.77	55.02	28.26	17.92	0.00	0.00	0.00	0.00	0.00	0.00	8.59	0.00	3.98	0.00	0.00	0.00	0.00	0.00	0.00
Chrnb2	78.90	23.43	134.51	25.77	43.13	31.83	15.83	13.12	24.99	83.16	21.37	99.08	55.77	35.06	53.54	75.05	92.14	50.69	79.18
Chrnb3	43.53	0.46	138.25	5.91	0.00	0.00	0.00	0.00	0.00	0.00	0.00	0.00	4.86	0.00	0.00	0.00	0.00	0.00	0.00
Chrnb4	4.68	1.14	2.02	1.33	0.00	0.00	0.00	0.00	0.00	0.00	0.00	0.00	0.00	0.00	0.00	0.00	0.00	0.00	0.00
Chrnd	0.00	0.00	0.00	0.00	0.00	0.00	0.00	0.00	0.00	0.00	0.00	0.00	0.00	0.00	0.00	0.00	0.00	0.00	0.00
Chrne	0.00	0.00	0.00	0.00	0.00	0.00	0.00	0.00	0.00	0.00	0.00	0.00	0.00	0.00	0.00	0.00	0.00	0.00	0.00
Chrng	0.00	0.00	0.00	0.00	0.00	0.00	0.00	0.00	0.00	0.00	0.00	0.00	0.00	0.00	0.00	0.00	0.00	0.00	0.00

	Ventral midbrain					Spiral ganglion			Inner ear hair cells			
	SNC	VTA1	VTA2	VTA3	VTA4	TypeIa	TypeIb	TypeIc	Utricle_HC_P1	Utricle_HC_P12	Utricle_HC_P100	Cochlear_HC
Chrna1	0.00	0.00	0.00	0.00	0.00	76.33	5.52	27.55	320.99	376.92	388.80	154.91
Chrna2	0.00	0.00	0.00	0.00	0.00	0.00	0.00	0.00	0.00	0.00	0.00	0.00
Chrna3	75.04	61.02	34.75	0.00	9.22	0.00	0.00	16.67	0.00	0.00	0.00	0.00
Chrna4	559.44	674.74	277.56	568.53	274.87	131.75	57.37	18.66	0.00	0.00	0.00	0.00
Chrna5	28.65	16.99	19.36	0.00	3.32	0.00	0.00	0.00	0.00	0.00	0.00	0.00
Chrna6	1286.75	718.59	1140.92	705.52	821.02	0.00	0.00	11.64	0.00	0.00	0.00	0.00
Chrna7	0.00	0.00	0.00	0.00	0.00	37.61	49.86	21.04	0.00	0.00	0.00	0.00
Chrna9	0.00	0.00	0.00	0.00	0.00	15.33	13.61	0.00	554.41	626.98	607.56	350.02
Chrna10	0.00	0.00	0.00	0.00	0.00	0.00	4.42	7.33	464.02	632.15	599.18	466.89
Chrnb1	0.00	0.00	0.00	0.00	0.00	80.87	59.75	76.27	15.00	39.59	26.26	39.70
Chrnb2	37.12	100.00	91.58	117.53	70.01	298.16	295.47	315.08	96.02	175.77	182.60	62.75
Chrnb3	326.52	370.87	260.30	351.18	344.16	0.00	3.64	11.32	0.00	0.00	0.00	0.00
Chrnb4	0.00	0.00	0.00	0.00	0.00	4.39	18.07	0.00	24.11	22.30	2.31	4.21
Chrnd	0.00	0.00	0.00	0.00	0.00	0.00	0.00	0.00	0.00	0.00	0.00	2.35
Chrne	0.00	0.00	0.00	0.00	0.00	4.97	5.00	7.81	0.00	5.92	22.78	0.00
Chrng	0.00	0.00	0.00	0.00	0.00	0.00	0.00	0.00	509.75	40.42	0.00	32.97

	Visceral motor neurons								Dorsal root ganglia										
	Nor1	Nor2	Nor3	Nor4	Nor5	Glut	Ach1	Ach2	NF1	NF2	NF3	NF4	NF5	NP1	NP2	NP3	PEP1	PEP2	TH
Chrna1	0.00	0.00	0.00	0.00	0.00	0.00	0.00	0.00	0.00	0.00	15.18	0.00	0.00	0.00	0.00	0.00	0.00	0.00	0.79
Chrna2	0.00	0.00	0.00	0.00	0.00	0.00	0.00	0.00	0.00	0.00	0.00	0.00	0.00	0.00	0.00	0.00	0.00	0.00	0.00
Chrna3	535.34	689.45	825.02	808.62	988.48	107.72	572.22	596.98	0.00	0.00	0.00	0.00	0.00	0.00	0.00	0.00	0.00	40.86	0.00
Chrna4	0.00	0.00	0.00	0.00	0.00	0.00	9.93	0.00	0.00	3.45	0.00	0.00	0.00	0.00	0.00	44.71	0.00	0.00	28.78
Chrna5	55.99	50.79	41.92	38.86	38.83	0.00	51.51	37.33	0.00	0.00	0.00	0.00	0.00	0.00	0.00	0.00	0.00	0.00	0.00
Chrna6	0.00	0.00	0.00	0.00	0.00	79.15	0.00	0.00	76.83	4.69	0.00	0.00	0.00	95.66	128.57	126.53	89.34	0.00	6.40
Chrna7	12.13	30.63	39.57	15.58	9.06	0.00	0.00	8.86	0.00	0.00	9.14	0.00	0.00	0.00	0.00	0.00	0.00	15.08	0.00
Chrna9	0.00	0.00	0.00	0.00	0.00	0.00	0.00	0.00	0.00	0.00	0.00	0.00	0.00	0.00	0.00	0.00	0.00	0.00	0.00
Chrna10	0.00	0.00	0.00	0.00	0.00	0.00	0.00	0.00	0.00	0.00	3.03	0.00	0.00	0.00	0.00	0.00	0.00	17.27	0.00
Chrnb1	0.00	0.00	0.00	0.00	0.00	0.00	7.76	0.00	0.00	0.00	0.00	0.00	0.00	0.00	0.00	0.00	11.97	0.00	0.00
Chrnb2	90.59	50.45	60.37	46.26	56.20	42.76	62.82	61.41	62.69	45.23	134.24	25.43	61.54	79.49	21.35	59.17	25.69	50.66	47.42
Chrnb3	0.00	0.00	0.00	0.00	0.00	109.59	0.00	0.00	0.00	0.00	0.00	0.00	0.00	4.15	0.00	0.00	9.39	0.00	1.70
Chrnb4	154.87	202.31	264.13	290.53	232.07	42.76	222.23	164.63	0.00	0.00	0.00	0.00	0.00	1.81	0.00	0.00	0.00	23.00	0.00
Chrnd	0.00	0.00	0.00	0.00	0.00	0.00	0.00	0.00	0.00	0.00	0.00	0.00	0.00	0.00	8.76	0.00	0.00	0.00	0.00
Chrne	0.00	0.00	0.00	0.00	0.00	0.00	0.00	0.00	0.00	0.00	0.00	0.00	0.00	0.00	0.00	0.00	0.00	0.00	0.00
Chrng	0.00	0.00	0.00	0.00	0.00	0.00	0.00	0.00	0.00	0.00	0.00	0.00	0.00	0.00	0.00	0.00	0.00	0.00	0.00

Values are means of the estimated joint posterior distributions of gene expression levels inferred for the nAChR subunits expressed in the cell types and datasets analysed.

Supplementary table S6. Neuronal nAChRs experimentally validated assemblies.

nAChR subtype	DOI	Reference
α7	10.1016/0896-6273(90)90344-F	Couturier et al., 1990
α8	PMID: 7509438	Gerzanich et al., 1994
α9	10.1016/0092-8674(94)90555-X	Elgoyhen et al., 1994
α10	10.1093/molbev/msu258	Lipovsek et al., 2014
α2β2	10.1016/0896-6273(88)90208-5	Deneris et al., 1988
α2β4	10.1016/0896-6273(89)90207-9	Duvoisin et al.,1989
α3β2	10.1073/pnas.84.21.7763	Boulter et al., 1987
α3β4	10.1016/0896-6273(89)90207-9	Duvoisin et al.,1989
α4β2	10.1073/pnas.84.21.7763	Boulter et al., 1987
α4β4	10.1016/0896-6273(89)90207-9	Duvoisin et al., 1989
α6β2	10.1046/j.1460-9568.1998.00001.x	Fucile et al., 1998
α6β4	10.1124/mol.106.027326	Tumkosit et al., 2006
α7β2	10.1113/jphysiol.2001.013847	Khiroug et al., 2002
α7β3	10.1074/jbc.274.26.18335	Palma etal., 1999
α7β4	110.1111/j.1471-4159.2012.07931.x	Criado et al., 2012
α9α10	10.1073/pnas.051622798	Elgoyhen et al., 2001
α2α5β2	10.1124/mol.63.6.1329	Vailati et al., 2003
α2α6β2	10.1124/mol.105.015925	Gotti et al., 2005
α2β2β3	10.1111/j.1471-4159.2012.07685.x	Dash et al., 2012
α2β3β4	10.1111/j.1471-4159.2012.07685.x	Dash et al., 2012
α3α4β2	10.1523/JNEUROSCI.2112-05.2005	Turner et al., 2005
α3α4β4	10.1523/JNEUROSCI.2112-05.2005	Turner et al., 2005
α3α5β2	10.1074/jbc.271.30.17656	Wang et al., 1996
α3α5β4	10.1074/jbc.271.30.17656	Wang et al., 1996
α3α6β2	10.1016/S0028-3908(00)00144-1	Kuryatov et al., 2000
α3α6β4	10.1046/j.1460-9568.1998.00001.x	Fucile et al., 1998
α3β2β4	10.1523/JNEUROSCI.2112-05.2005	Turner et al., 2005
α3β2β3	10.1111/j.1471-4159.2012.07685.x	Dash et al., 2012
α3β3β4	10.1074/jbc.273.25.15317	Groot-Kormelink et al., 1998
α4α5β2	10.1038/380347a0	Ramirez-Latorre et al., 1996
α4β2β3	10.1124/mol.108.046789	Kuryatov et al., 2008
α4β3β4	10.1111/j.1471-4159.2012.07685.x	Dash et al., 2012
α5α6β2	10.1016/S0028-3908(00)00144-1	Kuryatov et al., 2000
α5α7β2	10.1111/j.1749-6632.1999.tb11331.x	Girod et al., 1999
α6β2β3	10.1124/mol.106.027326	Tumkosit et al., 2006
α6β3β4	10.1016/S0028-3908(00)00144-1	Kuryatov et al., 2000
α3α4α6β2	10.1111/j.1471-4159.2008.05282.x	Cox etal., 2008
α3α5β2β4	10.1074/jbc.270.9.4424	Conroy and Berg, 1995
α4α6β2β3	10.1124/mol.110.066159	Kuryatov and Lindstrom, 2010
α5α6β3β4	10.1124/jpet104.075069	Grinevich et al., 2005
α1β1γδ	10.1038/321406a0	Mishina et al., 1986
α1β1δε	10.1038/321406a0	Mishina et al., 1986

Experimentally validated pentameric combinations of nAChR subunits and relevant literature references.

Supplementary table S7. Primers used for the cloning of *X. tropicalis* nAChRs subunits.

Primer	Tm (ºC)	Sequence (5'-3')
**FXtrop.a9**	61.4	TA**GAATTC**ATGCACACATTTTTGGCACGTGTTGG
**RXtrop.a9**	63.1	AT**CTCGAG**TCACACTGCCTGGGCAATAA
**FXtrop.a10**	62.4	AT**AAGCTT**TCCTGCCCAACATGAGACTGC
**RXtrop.a10**	62.1	AT**CTCGAG**GGCACCATCATATGGCTTTG
**FXtrop.a7**	61.8	TA**GAATTC**TTTGATCTCCTGCGATGGGAGGA
**RXtrop.a7**	61.9	AT**CTCGAG**TAGAACTGCAGAACCCTAAGCAA
**FXtrop.a4**	62.7	AT**GAATTC**GCTCTCTGCCAACATGGGTG
**RXtrop.a4**	60.1	TA**AAGCTT**GGGGTCAGTTCATATCAAACCAG
**FXtrop.b2**	61.2	TA**GAATTC**GAGGCGATGAGATGATCCGG
**RXtrop.b2**	61.3	AT**CTCGAG**GCCCTCAATGTTTCTCAGTT

The restriction sites for EcoRI, XhoI and HindIII used for subsequent subcloning are highlighted in bold. Tm, annealing temperature.

Supplementary table S8. Normalized biophysical properties

Receptor	ACh sensitivity (EC_{50})	Desensitization (%*I* after ACh peak)	Ca^{2+} modulation ($I_{0.5\ mM}/I_{3\ mM}$)	Ca^{2+} permeability (%*I* post-BAPTA)	Rectification ($I_{+40\ mV}/I_{-90\ mV}$)
**Rat α9α10**	0.045	0.830	1.000	0.248	0.525
**Chick α9α10**	0.041	0.783	0.266	1.000	1.000
**Frog α9α10**	0.257	0.187	0.167	0.195	0.091
**Rat α4β2**	0.007	0.993	0.097	0.719	0.029
**Chick α4β2**	0.002	1.000	0.146	0.785	0.014
**Frog α4β2**	0.002	0.936	0.158	0.813	0.008
**Rat α7**	1.000	0.042	0.167	0.290	0.016
**Chick α7**	0.619	0.041	0.121	0.370	0.009
**Frog α7**	0.554	0.026	0.168	0.384	0.015

**Amniote α4β2**	0.004	0.985	0.128	0.762	0.019
**Tetrapod α4β2**	0.003	0.963	0.141	0.785	0.014
**Amniote α7**	0.679	0.038	0.141	0.357	0.012
**Tetrapod α7**	0.675	0.037	0.142	0.358	0.012
**Amniote α9α10**	0.114	0.594	0.409	0.549	0.581
**Tetrapod α9α10**	0.139	0.522	0.366	0.487	0.494

Normalized biophysical properties from extant receptors used in PCA analysis (top) and normalised inferred biophysical properties from ancestral receptors. Values are normalised relative to the maximum obtained for each parameter.

Supplementary table S9. PCA analysis of functional properties.

	PC1	PC2	PC3	PC4	PC5
**ACh sensitivity (EC_{50})**	0.55	0.04	0.35	0.72	0.23
**Desensitization (%*I* after ACh peak)**	-0.56	-0.11	-0.35	0.41	0.62
**Ca^{2+} modulation ($I_{0.5\ mM}/I_{3\ mM}$)**	-0.18	0.77	-0.26	0.36	-0.42
**Ca^{2+} permeability (%*I* post-BAPTA)**	-0.46	-0.46	0.37	0.38	-0.55
**Rectification ($I_{+40\ mV}/I_{-90\ mV}$)**	-0.36	0.42	0.74	-0.21	0.31
**Proportion of the variability**	0.54	0.28	0.14	0.03	0.006
**Accumulated proportion**	0.54	0.82	0.96	0.99	1.00

Upper panel: loading of each experimental parameter on the five principal components. *Lowe panel*: proportion and accumulated proportion of the variability represented by each principal component.

Supplementary table S10. Estimated distances between extant and inferred ancestral α9α10, α4β2 and α7 nAChRs based on protein sequence identity.

**α4β2**		
**Comparison**	**seqID**	**1-seqID**
Rat-amniote	0.824	0.176
Chick-amniote	0.816	0.184
Amniote-tetrapod	0.819	0.181
Frog-tetrapod	0.777	0.223

**α7**		
**Comparison**	**seqID**	**1-seqID**
Rat-amniote	0.888	0.112
Chick-amniote	0.962	0.038
Amniote-tetrapod	0.997	0.003
Frog-tetrapod	0.907	0.093

**α9α10**		
**Comparison**	**seqID**	**1-seqID**
Rat-amniote	0.683	0.317
Chick-amniote	0.821	0.179
Amniote-tetrapod	0.96	0.04
Frog-tetrapod	0.814	0.186

Sequence identity (seqID) values between pairs of sequences were calculated using the protein sequence alignments in Supplementary File 3). Distances (1-seqID values) were subsequently used as branch lengths for the receptor trees used for the inference of ancestral character states for each nAChR (Fig. S6).

Supplementary File Legends

Supplementary File 1. Multiple alignment of nAChRs subunits. The alignment includes 392 nAChR subunits sequences from 29 different species together with 9 sequences from 5HT3B vertebrate subunits (outgroup).

Supplementary File 2. Aminoacid sequence identities. Values for all pairwise comparisons for all nAChR subunits from amniotes.

Supplementary File 3. Type II functional divergence analysis using DIVERGE 3.0. For each nAChR subunit analysed, the main tree, cluster-mammalian tree and cluster-sauropsid tree are shown in parenthetical notation. Theta-II values and standard error (SE) were calculated by comparing the designated clusters. Posterior probabilities (Pp) per site were calculated from the Posterior ratio (Pr) values obtained from the type II function by *Pp = Pr / (1+Pr)*, as in Gu et al, 2006.

Supplementary File 4. Sequences used for the reconstruction of ancestral nAChR subunits. DNA sequences from α4, α7, α9, α10 and β2 orthologues used to reconstruct DNA sequences from amniote and tetrapod ancestors. The species trees, in parenthetic format, are shown before the sequence alignment, in fasta format. The branch lengths in the species trees were inferred from each alignment and indicate the amount of accumulated changes.

Supplementary File 5. Extant and inferred ancestral nAChRs protein sequences. Protein sequences from rat, chicken, frog, amniote ancestor and tetrapod ancestor, from α7, $(\alpha4)_2(\beta2)_3$ and $(\alpha9)_2(\alpha10)_3$ nAChRs were aligned and the sequence identity between pairs of receptors was used to calculate the branch lengths assigned to the receptor trees (Supplementary Figure 6) used to infer ancestral character states of biophysical properties (Supplementary Figure 7).
